# Anti-metabolite chemotherapy increases LAG-3 expressing tumor-infiltrating lymphocytes which can be targeted by combination immune checkpoint blockade

**DOI:** 10.1136/jitc-2023-008568

**Published:** 2024-09-28

**Authors:** Nicola Principe, Amber-Lee Phung, Kofi L P Stevens, Omar Elaskalani, Ben Wylie, Caitlin M Tilsed, Fezaan Sheikh, M Lizeth Orozco Morales, Joel Kidman, Elly Marcq, Scott A Fisher, Anna K Nowak, Alison M McDonnell, W Joost Lesterhuis, Jonathan Chee

**Affiliations:** 1 Institute for Respiratory Health, National Centre for Asbestos Related Diseases, The University of Western Australia, Perth, Western Australia, Australia; 2 School of Biomedical Sciences, The University of Western Australia, Perth, Western Australia, Australia; 3 Telethon Kids Institute, Nedlands, Western Australia, Australia; 4 Perelman School of Medicine, Pulmonary, Allergy and Critical Care Division, University of Pennsylvania, Philadelphia, Pennsylvania, USA; 5 Center for Oncological Research (CORE), Integrated Personalized and Precision Oncology Network (IPPON), University of Antwerp, Antwerpen, Belgium; 6 Brussels Center for Immunology, Vrije Universiteit Brussel, Brussels, Belgium; 7 Lab of Dendritic Cell Biology and Cancer Immunotherapy, VIB Center for Inflammation Research, Brussels, Belgium; 8 Medical School, The University of Western Australia, Crawley, Western Australia, Australia

**Keywords:** Immune checkpoint inhibitors, T-lymphocytes, Chemotherapy, Combination therapy

## Abstract

**Background:**

Antibodies that target immune checkpoints such as cytotoxic T lymphocyte antigen 4 (CTLA-4), programmed cell death protein/ligand 1 (PD-1/PD-L1) are approved for treatment of multiple cancer types. Chemotherapy is often administered with immune checkpoint blockade (ICB) therapies that target CTLA-4 and/or PD-(L)1. ICB targeting other immune checkpoints such as lymphocyte activating gene-3 (LAG-3) has the potential to improve antitumor responses when combined with chemotherapy. Response to anti-PD-1 ICB is dependent on progenitor exhausted CD8^+^ T cells (T_PEX_) in the tumor, but it is unclear how chemotherapy alters T_PEX_ proportions and phenotype.

**Methods:**

Here we investigated whether sequential chemotherapy altered T_PEX_ frequency and immune checkpoint expression in multiple murine tumor models.

**Results:**

Two doses of two different anti-metabolite chemotherapies increased tumor infiltrating CD4^+^, and CD8^+^ T_PEX_ expressing LAG-3 in multiple mouse models, which was not restricted to tumor antigen specific CD8^+^ T cells. To determine if LAG-3^+^tumor infiltrating lymphocytes (TILs) could be targeted to improve tumor control, we administered anti-LAG-3 and anti-PD-1 ICB after two doses of chemotherapy and found combination therapy generated robust antitumor responses compared with each agent alone. Both anti-LAG-3 and anti-PD-1 ICB with chemotherapy were required for the complete tumor regression observed.

**Conclusions:**

Changes in immune checkpoint expression on TILs during chemotherapy administration informs selection of ICB therapies to combine with.

**WHAT IS ALREADY KNOWN ON THIS TOPIC**

- The efficacy of anti-programmed cell death protein 1 (PD-1) immune checkpoint blockade (ICB) is dependent on the differentiation and exhaustion state of CD8^+^ tumor infiltrating lymphocytes (TILs). Chemotherapy is often combined with ICB, but the effects of chemotherapy alone on CD8^+^ TIL exhaustion are not well described. Understanding these effects will help tailor effective chemotherapy and ICB combinations.

**WHAT THIS STUDY ADDS**

- Sequential doses of anti-metabolite chemotherapies increased frequencies of lymphocyte activating gene-3 (LAG-3) and PD-1 expressing progenitor exhausted CD8^+^ and CD4^+^ TILs in mesothelioma and colon cancer. The addition of LAG-3 and PD-1 blockade to chemotherapy led to improved survival in both models.

**HOW THIS STUDY MIGHT AFFECT RESEARCH, PRACTICE OR POLICY**

- Monitoring the tumor-immune milieu throughout chemotherapy administration could provide individualized selection of ICB therapies and may directly impact early phase clinical testing of anti-LAG-3/anti-PD-1 with chemotherapy.

## Background

 Immune checkpoint blockade (ICB) therapies that target cytotoxic T lymphocyte antigen 4 (CTLA-4), programmed cell death protein 1 (PD-1) or programmed cell death ligand 1 (PD-L1) have resulted in durable antitumor responses in a subset of cancer patients. ICB that target other immune checkpoints (eg, lymphocyte activating gene-3 (LAG-3), T-cell immunoglobulin and mucin-domain containing-3 (TIM-3), T-cell immunoreceptor with immunoglobulin and ITIM domain (TIGIT)), have been assessed in preclinical studies and are currently being trialed in patients across many cancer types.^1 2^ However, the effects of anti-TIM-3, anti-LAG-3 or anti-TIGIT ICB in combination with traditional anticancer treatment modalities such as chemotherapy remain unclear.

Chemotherapy remains the standard of care in a majority of cancers and is one of the most effective treatment modalities when combined with ICB therapy.^3^ Chemotherapy and anti-CTLA-4 and/or anti-PD-(L)1 ICB combinations have been successful for many hard-to-treat cancers, such as mesothelioma,^4^ non-small cell lung cancer,^5^ breast cancer^6^ and colorectal cancer.^7^ The synergistic effect of combination therapy is linked to the multiple immunostimulatory effects induced by chemotherapies in the tumor microenvironment.^8 9^ These include increased antigen presentation and dendritic cell activation,^10 11^ altered immune checkpoint expression,^12^ increased immunogenic cell death,^13^ depletion of immunosuppressive cells^14 15^ and increased activation and proliferation of tumor infiltrating lymphocytes (TILs) including CD8^+^ T cells.^16 17^

The differentiation state of tumor infiltrating CD8^+^ T cells is crucial for the success of ICB. Anti-PD-1 ICB acts on a subset of CD8^+^ T cells that retain proliferative and cytotoxic capacity despite being suppressed within the tumor microenvironment.^18^ This subset of CD8^+^ T cells exists in a progenitor exhausted (T_PEX_) differentiation state and is characterized by the expression of surface PD-1, SLAM family member 6 (SLAMF6) and transcription factor TCF1.^19^ In murine models, efficacy of anti-PD-1 ICB is dependent on intratumoral T_PEX_.^20 21^ TCF1^+^ CD8^+^ T cells with proliferative potential are associated with anti-PD-1 ICB response across multiple clinical studies.^22^,^25^ Despite chemotherapy having immunostimulatory effects, it is unclear how chemotherapy changes the differentiation state of CD8^+^ T cells, specifically the frequency and the expression levels of inhibitory checkpoint receptors on tumor infiltrating CD8^+^ T_PEX_. As many patients with cancer receive chemotherapy, understanding how chemotherapy affects CD8^+^ T cells could help guide clinical decisions by determining which ICB agent patients should receive in combination with chemotherapy in hope to achieve the best therapeutic response.

In this study, we characterized dynamic changes in CD8^+^ T_PEX_ after four chemotherapies in four murine tumor models and identified ICB targets (anti-PD-1/anti-LAG-3) to combine with chemotherapy. Anti-LAG-3 and anti-PD-1 ICB could be a beneficial treatment option to combine with anti-metabolite chemotherapy for cancers that currently have low response rates to ICB, like mesothelioma.

## Materials and methods

### Mice

BALB/c and C57BL/6 mice (RRID: IMSR_ARC:BC, RRID: IMSR_ARC:B6) were bred and maintained at the Animal Resources Centre (Murdoch, Western Australia, Australia) or Harry Perkins Institute of Medical Research (Murdoch and Nedlands, Western Australia, Australia). Clone 4 (CL4xThy1.1) T cell receptor (TCR) transgenic mice express a TCR that recognizes a major histocompatibility complex (MHC) class I-restricted influenza A/PR/8 hemagglutinin (HA_533−541_) epitope.^26^ CL4xThy1.1 mice were kindly provided by Professor Linda Sherman (The Scripps Research Institute, La Jolla, California, USA) and bred at the Harry Perkins Institute of Medical Research. All mice used were between 8 and 10 weeks of age, female and were maintained under standard, specific pathogen-free housing conditions at the Harry Perkins Bioresources North Facility (Nedlands, Western Australia, Australia).

### Cell lines

Cell lines AB1-HA, AE17 and CT26 were maintained in Roswell Park Memorial Institute Medium (RPMI) 1640 (Thermo Fisher Scientific, Scoresby Victoria, Australia) supplemented with 20 mM HEPES, 0.05 mM 2-Mercaptoethanol, 100 units/mL penicillin (CSL, Melbourne Victoria, Australia), 50 µg/mL gentamicin (David Bull Labs, Kewdale Victoria, Australia), 10% Newborn Calf Serum (NCS; Thermo Fisher Scientific, Scoresby Victoria, Australia) and 50 mg/mL of geneticin for AB1-HA only (G418; Life Technologies). MC38 was maintained in high glucose-pyruvate DMEM (Harry Perkins Institute, Nedlands Western Australia, Australia) supplemented with 10% NCS, 100 units/mL penicillin, 50 µg/mL gentamicin. Murine mesothelioma cell lines: AB1-HA (CBA-1374) and AE17 (CBA-0156) were derived as previously described.^27 28^ Murine colon cancer cell line: CT26 was obtained from ATCC (ATCCRL2638) and MC38 was obtained from Merck Sigma-Aldrich (SCC172). All cell lines were tested for *Mycoplasma* spp, every 3–4 months by PCR and found to be negative.

### Transfer of TCR transgenic splenocytes

Spleens from CL4xThy1.1 mice were manually dissociated through 40 µm strainers with phosphate-buffered saline (PBS) supplemented with 2% NCS (Life Technologies). Red blood cells were lysed with Pharm Lyse (BD Biosciences) and splenocytes were washed twice with PBS. Splenocytes (1×10^6^) were suspended in 100 µL of PBS and intravenously injected into mice 24 hours prior to tumor inoculation where described.

### Tumor cell inoculation

Tumor cells were harvested when they reached 80% confluence after a minimum of three passages after thawing. The right-hand flanks of mice were inoculated subcutaneously with 5×10^5^ tumor cells suspended in 100 µL of PBS. Mice were randomized prior to treatment, when tumor were palpable. Tumor dimensions (length and width) were measured with calipers and growth was represented as area (mm^2^). The investigator making tumor measurements was blinded to the treatment group for survival experiments.

### Chemotherapy and immune checkpoint blockade therapy

Mice were administered with two doses in 3-day intervals of gemcitabine (GEM; 240 mg/kg), 5-fluorouracil (5FU; 30 mg/kg), cyclophosphamide (CTX; 100 mg/kg) or cisplatin (CP; 4 mg/kg) chemotherapies. Each single dose is less than half the previously determined maximum tolerated dose.^29 30^ Chemotherapies were provided by Sir Charles Gairdner Pharmacy (Nedlands, Western Australia, Australia). Anti-PD-1 (clone RMP1-14, Bio X Cell) and anti-LAG-3 (clone C9B7W, Bio X Cell) were dosed three times with 2-day intervals at 100 µg/mouse. All treatments were diluted in sterile 0.9% sodium chloride and administered intraperitoneally, except 5FU which was administered intravenously. Control mice received PBS at the equivalent volume.

### Preparation of single cell suspensions

Tumor draining axillary and inguinal draining lymph nodes (DLN) were manually dissociated through 40 µm strainers with PBS supplemented with 2% NCS (Life Technologies). Tumors were processed using 1.5 mg/mL type IV collagenase (Worthington Biochemical) and 0.1 mg/mL type I DNAse (Sigma-Aldrich) in PBS+2% NCS for 1 hour at 37°C on a Microtitre Plate Shaker Incubator (Thomas Scientific) as previously described.^31^ Cell counts were performed using a hemocytometer with trypan blue exclusion.

### Dendritic cell T-cell co-culture

Dendritic cell (DC) T-cell co-culture was set-up as previously described.^10^ Briefly, DCs were isolated from PBS or GEM-treated AB1-HA tumors using CD11c MicroBeads (Miltenyi Biotec). DCs were also purified from a spleen from a PBS-treated animal. Splenic DCs were pulsed with HA peptide (1 µg/mL) in RPMI with 20% fetal bovine serum (FBS) for 30 min at 37°C and washed three times in RPMI+FBS. CD8^+^ T cells were isolated from naïve BALB/c and CL4xThy1.1 mice spleens using a CD8a^+^ T Cell Isolation Kit (Miltenyi Biotec) before being labeled with carboxyfluorescein succinimidyl ester (CFSE, 2.5 µM/mL/10^7^ cells) in PBS+0.1% bovine serum albumin for 10 min at 37°C. CFSE-labeled CD8^+^ T cells were washed twice with RPMI+FBS. Cell numbers were determined using a hemocytometer with trypan blue exclusion. CFSE-labeled CD8^+^ T cells were added to serial dilutions of tumor DCs or splenic DCs in 200 µL of R10+FBS in 96-well U-bottom plates and incubated for 60 hours at 37°C. Cultures were washed twice with RPMI+FBS before flow cytometry analysis.

### Flow cytometry

Flow cytometry panels outlined in online supplemental table S1 were used to characterize T-cell subsets. Zombie UV (BioLegend) viability dye was diluted in PBS and added to samples prior to surface antigen staining. All antibodies for surface staining were diluted in PBS+2% NCS. Cells were permeabilized using the Foxp3/Transcription Factor Staining Buffer Set (eBioscience). Cells were washed with Permeabilization Buffer (eBioscience) and subjected to intracellular staining. To stain for CD107a and interferon (IFN-γ), samples were subjected to CD107a FITC in R10 for 1 hour at 37°C. PMA (20 ng/mL), ionomycin (1 µg/mL), brefeldin A (1:1,000) and monensin (1:1,000) were added and incubated for an additional 4 hours at 37°C. Samples were washed twice with PBS+2% NCS prior to flow cytometry antibody staining. Single stain and fluorescence minus-one controls were also performed. Data was acquired using a BD LSRFortessa SORP or BD FACSymphony A5SE with 20,000 T-cell events collected per sample where possible. All flow cytometry analyses were completed using FlowJo Software V.10 (BD Biosciences). A summary of antibody concentrations and gating strategies are outlined (online supplemental table S1 and figure S1).

### Gene Set Enrichment Analysis

Whole transcriptome data from patients with breast cancer published in Park and colleagues^32^ was downloaded from the Gene Expression Omnibus (GSE123845). Gene Set Enrichment Analysis (GSEA),^33^ using published CD8^+^ T-cell exhaustion gene sets^34 35^ was performed on provided normalized transcriptome data (transcripts per million) from 68 paired pretreatment (T1) and on-treatment (T2) tumor biopsies or 115 T1 and 88 T2 unmatched tumor biopsies. Gene sets enriched with false discovery rates (FDR) of <0.25 were considered significant. A total of 1,000 permutations were performed, and all other default parameters were used.

### Statistical analysis

Data are presented as mean±SD. For flow cytometry experiments, statistical analyses were performed using two-way analysis of variance with Tukey’s multi-comparisons to compare the interaction between chemotherapy and PBS groups across two time points or between chemo-immunotherapy and monotherapy controls. Kaplan-Meier method was used for survival analysis with log-rank test (Mantel-Cox) to analyze significance. All statistics were performed using GraphPad Prism Software (GraphPad Software, RRD:SCR_002798, V.8). Results were significant when p<0.05 (*p<0.05, **p<0.01, ***p<0.001, ****p<0.0001).

## Result

### Multiple chemotherapy doses increased SLAMF6^+^PD-1^+^ CD8^+^ T cells in the tumor microenvironment

**Figure 1:**
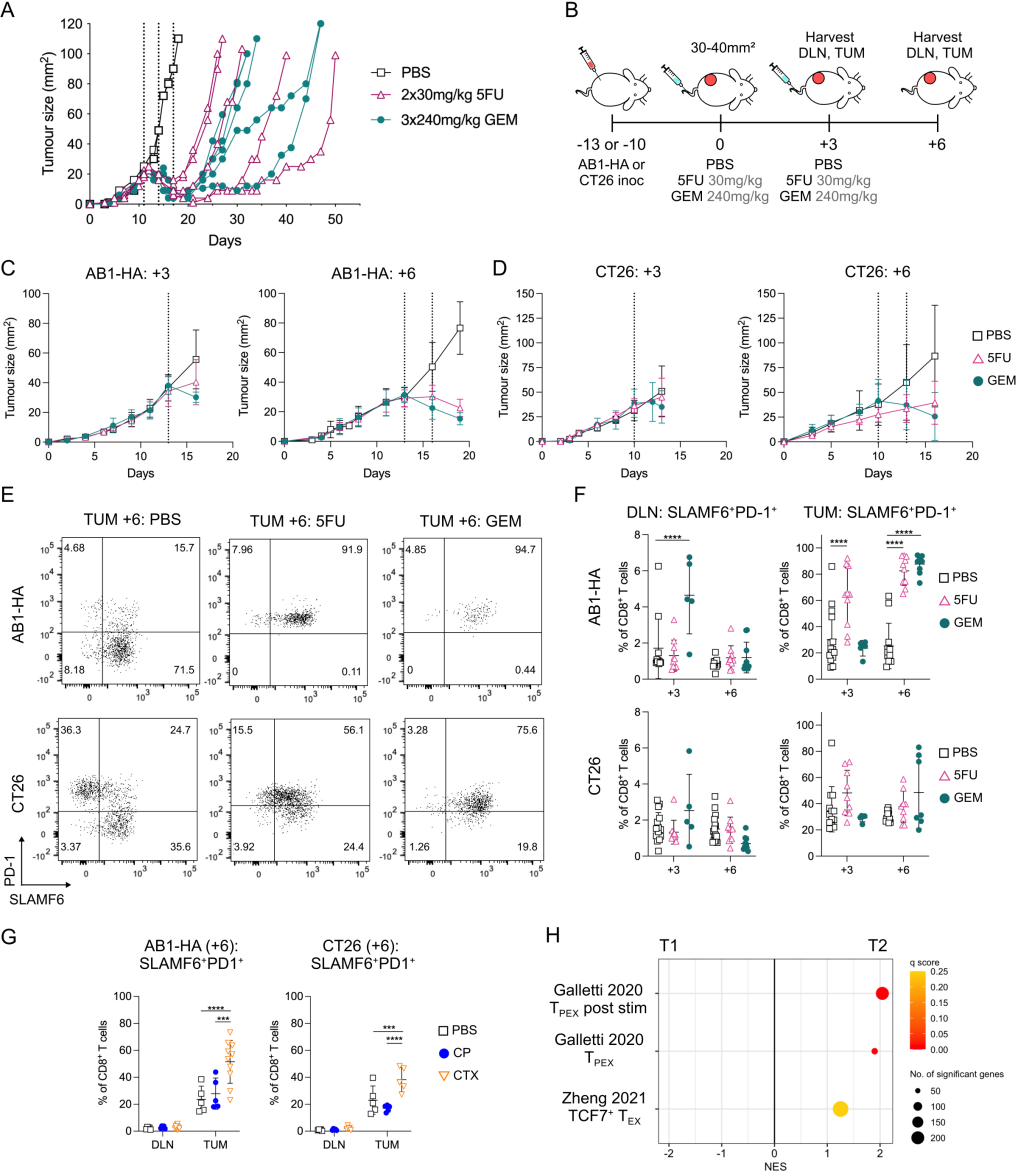
SLAMF6^+^PD-1^+^ CD8^+^ T_PEX_ increased in AB1-HA tumors after two doses of chemotherapy. (**A**) Representative tumor growth curves of AB1-HA tumor bearing mice treated with three doses of 240 mg/kg gemcitabine (GEM; n=4), two doses of 30 mg/kg 5-fluorouracil (5FU; n=4) chemotherapies or PBS (n=2). The first dose of chemotherapy was administered when tumors were 20–25 mm^2^, 11 days post tumor inoculation. (**B**) Experiment timeline. AB1-HA or CT26 tumor bearing animals were treated with 5FU, GEM or PBS when tumors reached 30–40 mm^2^ in size. Tumors (TUM) and tumor draining lymph nodes (DLN) were harvested for flow cytometry 3 days after either one dose (+3) or two doses of chemotherapy (+6). (**C–D**) Mean tumor growth curves of flow cytometry experiments for AB1-HA (**C**) and CT26 (**D**). Dotted lines indicate administration of chemotherapy. (**E**) Representative flow cytometry plots displaying SLAMF6^+^PD-1^+^ CD8^+^ T_PEX_ after two doses of chemotherapy (+6) in each treatment group of AB1-HA (top) and CT26 (bottom) tumors. (**F**) Dot plots representing frequencies of SLAMF6^+^PD-1^+^ CD8^+^ T_PEX_ in DLN (left) and tumors (right) in AB1-HA (top) and CT26 (bottom) after one (+3) or two (+6) doses of 5FU or GEM chemotherapy. (**G**) Dot plots showing proportion of SLAMF6^+^PD-1^+^ CD8^+^ T_PEX_ in DLN and tumors in AB1-HA (left) and CT26 (right) after two (+6) doses of cisplatin (CP; 2×4 mg/kg) or cyclophosphamide (CTX; 2×100 mg/kg) chemotherapy. (**H**) Gene Set Enrichment Analysis plots displaying CD8^+^ T_PEX_ gene sets significantly enriched (q<0.25) in on-treatment biopsies (T2) compared with pretreatment (T1) treatment biopsies from 68 patients with breast cancer.^32^ Flow cytometry data represented as mean±SD. Two-way analysis of variance with Tukey’s multiple comparisons test was used to compare between treatment groups and time points. Sample sizes for flow cytometry experiments were n=5–10 per treatment group, two pooled experiments. ****p≤0.0001, ***p<0.001. PBS, phosphate-buffered saline; PD-1, programmed cell death protein 1; SLAMF6, SLAM family member 6; T_PEX_, progenitor exhausted CD8^+^ T cells.

As T_PEX_ are key to anti-PD-1 ICB efficacy in murine cancer models,^20 21^ we investigated whether chemotherapy changed the frequency of CD8^+^ T_PEX_. We first tested this in two tumor models, AB1-HA mesothelioma and CT26 colon cancer, in which two sequential doses of 5FU or three doses of GEM chemotherapy delayed tumor growth, without being curative (figure 1A, online supplemental figure S2A). Both drugs are classed as anti-metabolites and we first characterized CD8^+^ T cells after two doses of each chemotherapy.

To determine how chemotherapy changed CD8^+^ T_PEX_ frequency, we harvested tumor and tumor DLN of AB1-HA or CT26 bearing mice 3 days after one dose (day+3) or two doses (day+6) of chemotherapy (figure 1B). At both time points, tumor size and total cell counts were greater in PBS-treated compared with 5FU and GEM treated animals (figure 1C,D, online supplemental figure S2B, C). Proportions of CD45^+^ cells and CD8^+^ T cells in tumors and DLNs were similar between groups at both time points (online supplemental figure S2D,E), allowing us to compare CD8^+^ T_PEX_ between treatment groups. We used SLAMF6 and PD-1 to mark CD8^+^ T_PEX_ as TCF1 and SLAMF6 were co-expressed on CD8^+^ T cells with and without chemotherapy, as previously reported (figure 1E, online supplemental figure S2F).

The proportion of CD8^+^ T_PEX_ (SLAMF6^+^PD-1^+^) significantly increased in DLNs after one dose of GEM (day+3: p≤0.0001) and in tumors after one dose of 5FU (day+3: p≤0.0001) compared with PBS controls in the AB1-HA model (figure 1F). There was no significant difference between chemotherapy and PBS-treated DLNs and tumors after one dose of chemotherapy in the CT26 model (figure 1F).

Two doses of chemotherapy (day+6) in AB1-HA tumor bearing animals significantly increased the frequency of tumor-infiltrating CD8^+^ T_PEX_ from 25.3±17.3% in PBS-treated animals to 82.6±11.1% (p≤0.0001) and 87.6±6.95% (p≤0.0001) in 5FU and GEM-treated animals, respectively (figure 1E,F). However, an increase of CD8^+^ T_PEX_ after two doses of chemotherapy was only observed in some tumors but not others for CT26-bearing animals, and overall there was no significant difference in CD8^+^ T_PEX_ frequency between PBS and chemotherapy treated tumors for this model. For both tumor models, there was no difference in CD8^+^ T_PEX_ proportions in DLNs (figure 1F), or total cell numbers (online supplemental figure S2G) after two doses of chemotherapy. We also characterized SLAMF6^−^PD-1^+^ CD8^+^ T cells, as the loss of SLAMF6 expression on CD8^+^ T cells is correlated with cells that are further differentiated, and have reduced proliferative function compared with CD8^+^ T_PEX_. In the AB1-HA model, the frequency of SLAMF6^−^PD-1^+^ CD8^+^ T cells in chemotherapy treated tumors was similar to PBS treated tumors (online supplemental figure S2H). In the CT26 model, two doses of GEM (day+6) significantly decreased the proportion of SLAMF6^−^PD-1^+^ CD8^+^ T cells compared with PBS controls (online supplemental figure S2H). In both models, the frequency of Ki67^+^ SLAMF6^+^PD-1^+^ CD8^+^ T_PEX_ cells was greater compared with Ki67^+^SLAMF6^−^PD-1^+^ CD8^+^ T cells after chemotherapy, highlighting the proliferative capacity of T_PEX_ (online supplemental figure S2I). These data suggest that intratumoral CD8^+^ T_PEX_ increased after two doses of anti-metabolite chemotherapy, mostly in the AB1-HA model.

To assess whether the increase in tumor-infiltrating CD8^+^ T_PEX_ extended to other chemotherapy classes apart from anti-metabolites, we characterized CD8^+^ T_PEX_ after two doses (+6) of cisplatin (CP) or cyclophosphamide (CTX) in both AB1-HA and CT26 (online supplemental figure S2J). Both chemotherapies are DNA alkylating agents. Two doses of CTX, but not CP significantly increased CD8^+^ T_PEX_ proportions in tumors compared with PBS controls in both AB1-HA (p≤0.0001) and CT26 (p=0.009) models (figure 1G). Proportions of CD8^+^ T_PEX_ in DLNs were similar between CTX or CP and PBS-treated animals (figure 1G). This data suggests that an increase in intratumoral CD8^+^ T_PEX_ was observed with a different chemotherapy in our models.

We next investigated whether CD8^+^ T_PEX_ gene signatures were enriched in patients with cancer undergoing chemotherapy. We performed GSEA on available tumor gene expression (bulk RNA sequencing) data from matched pretreatment (T1) and on-treatment (T2) biopsies from 68 patients with breast cancer treated with doxorubicin and CTX.^32^ Importantly, on-treatment (T2) biopsies were sampled at a fixed time point (3 weeks) after the initiation of chemotherapies, allowing us to evaluate the effects of chemotherapies on tumor immune milieu. Well-characterized CD8^+^ T_PEX_ gene sets^34 35^ were significantly enriched in T2 compared with T1 matched samples (figure 1H), with key T_PEX_ genes such as *PDCD1*, *SLAMF6* and *TCF7* upregulated. Genes encoding for inhibitory checkpoint receptors such as *CTLA4, HAVCR2, LAG3, TIGIT*, were also upregulated in T2 (online supplemental figure S2K). Enrichment of T_PEX_ gene signatures were also observed when the larger cohort of unmatched 112 T1 and 88 T2 samples were compared (online supplemental figure S2L). Data from animal models and clinical samples suggests that CD8^+^ T_PEX_ increased after certain chemotherapies.

### Anti-metabolite chemotherapy enhanced effector function and LAG-3 expression on intratumoral SLAMF6^+^PD-1^+^ CD8^+^ T_PEX_

**Figure 2:**
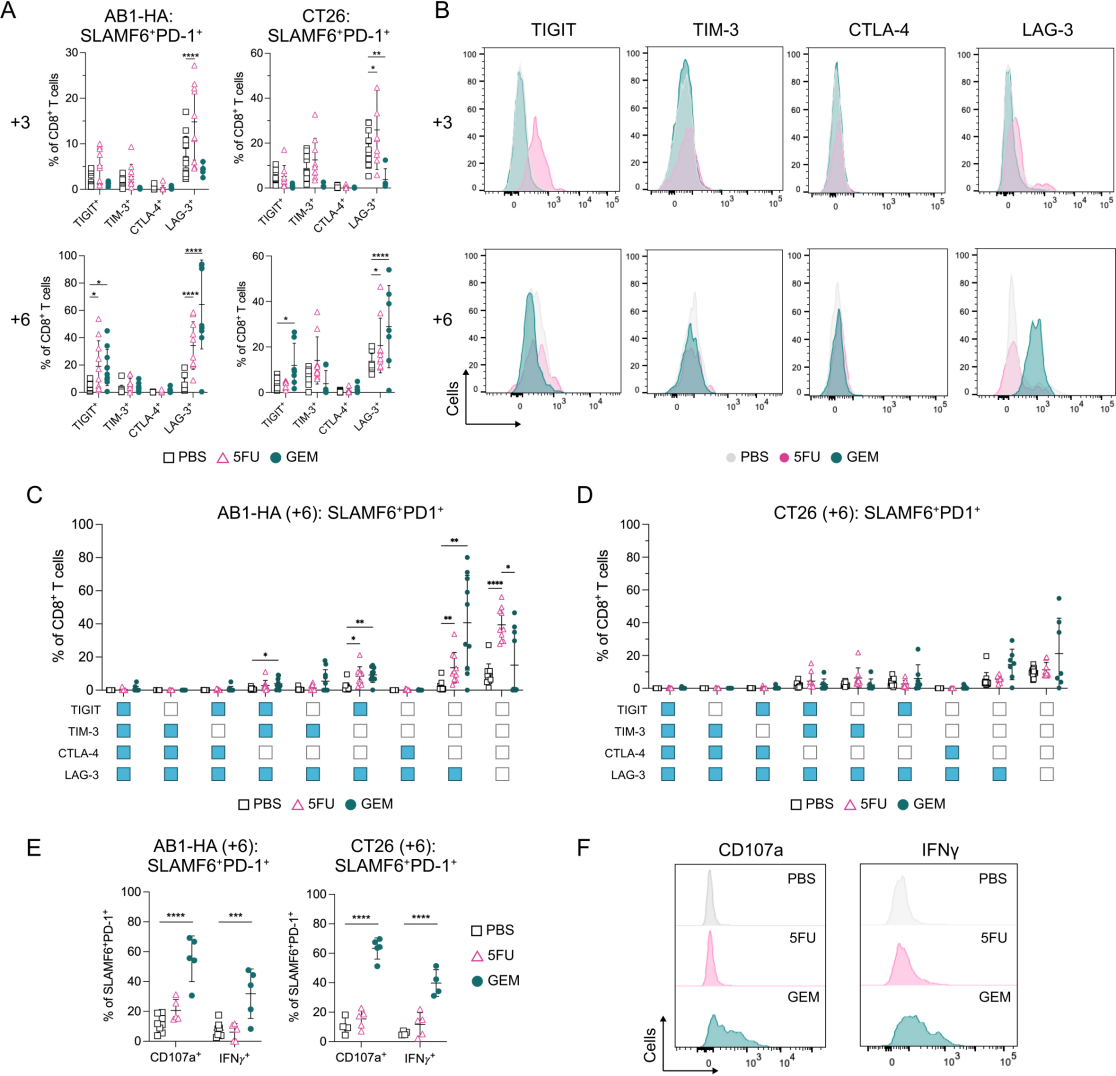
Sequential chemotherapy increases LAG-3 expression on SLAMF6^+^PD-1^+^ CD8^+^ T_PEX_ in AB1-HA and CT26 tumors. (**A**) Dot plots showing frequencies of TIGIT, TIM-3, CTLA-4 and LAG-3 expressing SLAMF6^+^PD-1^+^ CD8^+^ T_PEX_ in AB1-HA (left) and CT26 (right) tumors after one (+3; top) or two (+6; bottom) doses of chemotherapy. (**B**) Representative histograms comparing TIGIT, TIM-3, CTLA-4 and LAG-3 on SLAMF6^+^PD-1^+^ CD8^+^ T_PEX_ between PBS, 5FU and GEM chemotherapy in AB1-HA tumors after one (+3; top) or two (+6; bottom) doses of treatment. (**C–D**) Dot plots demonstrating frequencies of co-expression of LAG-3 with TIGIT, TIM-3 and CTLA-4 -on SLAMF6^+^PD-1^+^ CD8^+^ T_PEX_ in AB1-HA (**C**) and CT26 (**D**) tumors after two doses of chemotherapy. Positive expression of receptor is denoted blue, negative expression is denoted white. (**E**) Dot plots showing proportion of CD107a and IFN-γ expression on SLAMF6^+^PD-1^+^ CD8^+^ T_PEX_ in AB1-HA (left) and CT26 (right) tumors after two (+6) doses of chemotherapy. (**F**) Representative histograms comparing CD107a and IFN-γ expression on SLAMF6^+^PD-1^+^ CD8^+^ T_PEX_ after two doses of PBS, 5FU or GEM chemotherapy in AB1-HA tumors. Data represented as mean±SD. Two-way analysis of variance with Tukey’s multiple comparisons test was used to compare between treatment groups and time points. Sample sizes for flow cytometry experiments were n=5–10 per treatment group. *p<0.05, **p<0.01, ***p<0.001, ****p≤0.0001. CTLA-4, cytotoxic T lymphocyte antigen 4; GEM, gemcitabine; IFN, interferon; LAG-3, lymphocyte activating gene-3; PBS, phosphate-buffered saline; PD-1, programmed cell death protein 1; SALMF6, SLAM family member 6; TIGIT, T-cell immunoreceptor with immunoglobulin and ITIM domain; TIM-3, T-cell immunoglobulin and mucin-domain containing-3; TPEX, progenitor exhausted CD8^+^ T cells; 5-FU, 5-fluorouracil.

We next investigated the expression of inhibitory checkpoint receptors on CD8^+^ T_PEX_ in 5FU or GEM-treated animals. We analyzed the surface expression of TIGIT, TIM-3, CTLA-4 and LAG-3 on CD8^+^ T_PEX_ in tumors and DLNs. After one dose of 5FU (day+3), the proportion of intratumoral LAG-3^+^ CD8^+^ T_PEX_ significantly increased compared with PBS treated tumors in both AB1-HA (PBS vs 5FU: 7.26±4.37 vs 14.8±8.37%; p≤0.0001) and CT26 (PBS vs 5FU: 17.1±7.52 vs 25.9±17.6%; p=0.01) (figure 2A,B, online supplemental figure S3A). The expression of LAG-3 was significantly lower after one dose of GEM compared with PBS (day+3) in CT26 (p=0.001) but not AB1-HA tumors (figure 2A,B, online supplemental figure S3A). In DLNs, the proportion of LAG3^+^CD8^+^ T_PEX_ significantly increased after one dose of GEM (day+3) compared with PBS controls in AB1-HA (p≤0.0001) but not CT26 (online supplemental figure S3B). The expression of TIGIT, TIM-3 and CTLA-4 were similar between PBS and chemotherapy (day+3) DLNs and tumors in both models (online supplemental figure S3B). These data indicate that one dose of 5FU increased LAG-3 expression on CD8^+^ T_PEX_ in both tumor models, which was not observed for GEM.

After two doses of 5FU (day+6), the expression of TIGIT significantly increased on CD8^+^ T_PEX_ in AB1-HA (p=0.01), but not CT26 tumors. Two doses of GEM (day+6) significantly increased the proportion of CD8^+^ T_PEX_ expressing TIGIT in both AB1-HA and CT26 tumors compared with PBS treated tumors (AB1-HA: p=0.02; CT26: p=0.04; figure 2A,B). The frequency of LAG-3 expressing CD8^+^ T_PEX_ significantly increased to over 30% after two doses of 5FU or GEM compared with 5% in PBS controls in both AB1-HA (PBS vs 5FU: p≤0.0001; PBS vs GEM: p≤0.0001) and CT26 (PBS vs 5FU: p=0.04; PBS vs GEM: p≤0.0001) models (figure 2A,B). These results were also confirmed by mean fluorescence intensity (MFI) measurements of LAG-3 on CD8^+^ T_PEX_ (figure 2B, online supplemental figure S3A). Frequencies of CD8^+^ T_PEX_ expressing these inhibitory receptors in DLNs after two doses of chemotherapy were similar to PBS controls (online supplemental figure S3B).

Next, we sought to define co-expression of multiple inhibitory checkpoint receptors after chemotherapy, as co-expression is a key feature of T cells that may differentiate into terminally exhausted CD8^+^ T cells in the tumor.^19^ As we observed differences in LAG-3 expression on CD8^+^ T_PEX_ after two doses of 5FU and GEM in both models, we focused on co-expression of LAG-3 with other inhibitory checkpoint receptors. In AB1-HA tumors, two doses of 5FU and GEM increased LAG-3^+^TIGIT^+^CD8^+^ T_PEX_ compared with PBS controls (PBS vs 5FU: p=0.014; PBS vs GEM: p=0.001; figure 2C). Frequencies of CD8^+^ T_PEX_ in GEM and 5FU treated tumors expressing only LAG-3 were significantly increased compared with PBS treated tumors (5FU: p=0.008; GEM: p=0.005; figure 2C). CD8^+^ T_PEX_ expressing PD-1 alone was significantly increased in 5FU compared with PBS treated TUM (PBS vs 5FU: 9.59±6.31 vs 39.4±9.95%; p≤0.0001; figure 2C). In 5FU or GEM treated CT26 tumors, there was no significant difference in frequencies of any CD8^+^ T_PEX_ populations that co-expressed LAG-3 with other checkpoint receptors (figure 2D), suggesting that the overall increase in LAG-3 expressing CD8^+^ T_PEX_ (figure 2A) was not restricted to any subpopulations in CT26.

We also characterized inhibitory receptor expression on SLAMF6^−^PD-1^+^ CD8^+^ T cells. While there was a small number of SLAMF6^−^PD-1^+^ CD8^+^ T cells in chemotherapy treated tumors, two doses of GEM (day+6) increased LAG-3 expression compared with PBS controls in AB1-HA (p≤0.0001) but not in CT26 (p=0.98; online supplemental figure S3C). The expression of TIGIT, TIM-3 and CTLA-4 on SLAMF6^−^PD-1^+^ CD8^+^ T cells were similar between chemotherapy and PBS controls at both time points (online supplemental figure S3C).

Next, we investigated intratumoral CD8^+^ T_PEX_ effector function after chemotherapy. In both models, two doses of GEM, but not 5FU significantly increased the frequency of CD107a^+^ and IFN-γ^+^ CD8^+^ T_PEX_ compared with PBS controls (CD107a: p≤0.0001; IFN-γ: p=0.0003) (figure 2E,F). The proportions of LAG-3^+^ CD8^+^ T_PEX_ expressing CD107a, IFN-γ and Tbet increased in GEM treated tumors compared with controls (online supplemental figure S3D). The frequency of GzmB^+^ or CD137^+^ CD8^+^ T_PEX_ were similar between chemotherapy and PBS treated tumors (online supplemental figure S3E). Taken together, these data indicate that multiple doses of anti-metabolite chemotherapy increased the frequency of IFN-γ producing and LAG-3 expressing CD8^+^ T_PEX_ in the tumor.

### LAG-3^+^ CD8^+^ T_PEX_ increased by gemcitabine is not restricted to tumor-antigen specific CD8^+^ T cells in vivo

We previously reported that intratumoral DCs from GEM-treated animals increased tumor-antigen specific T-cell proliferation.^10^ We queried whether intratumoral DCs from GEM-treated animals increased LAG-3 expression on tumor-antigen specific T cells in vitro. We leveraged our AB1-HA model, which expressed a model neo-antigen (HA), by co-culturing DCs isolated from AB1-HA tumors with CD8^+^ TCR transgenic cells specific for HA_533–541_ antigen (CL4xThy1.1), or CD8^+^ T cells from wild-type, non-transgenic counterparts (online supplemental figure S4A). DCs from GEM treated tumors significantly increased the frequency of proliferating HA-specific CD8^+^ T cells that expressed SLAMF6, PD-1, LAG-3 compared with DCs from PBS treated tumors (p=0.0051), but not when co-cultured with wild-type CD8^+^ T cells (online supplemental figure S4B–D). These data suggest that GEM can enhance antigen-specific CD8^+^ T_PEX_ expressing LAG-3 through DC mediated mechanisms.

**Figure 3:**
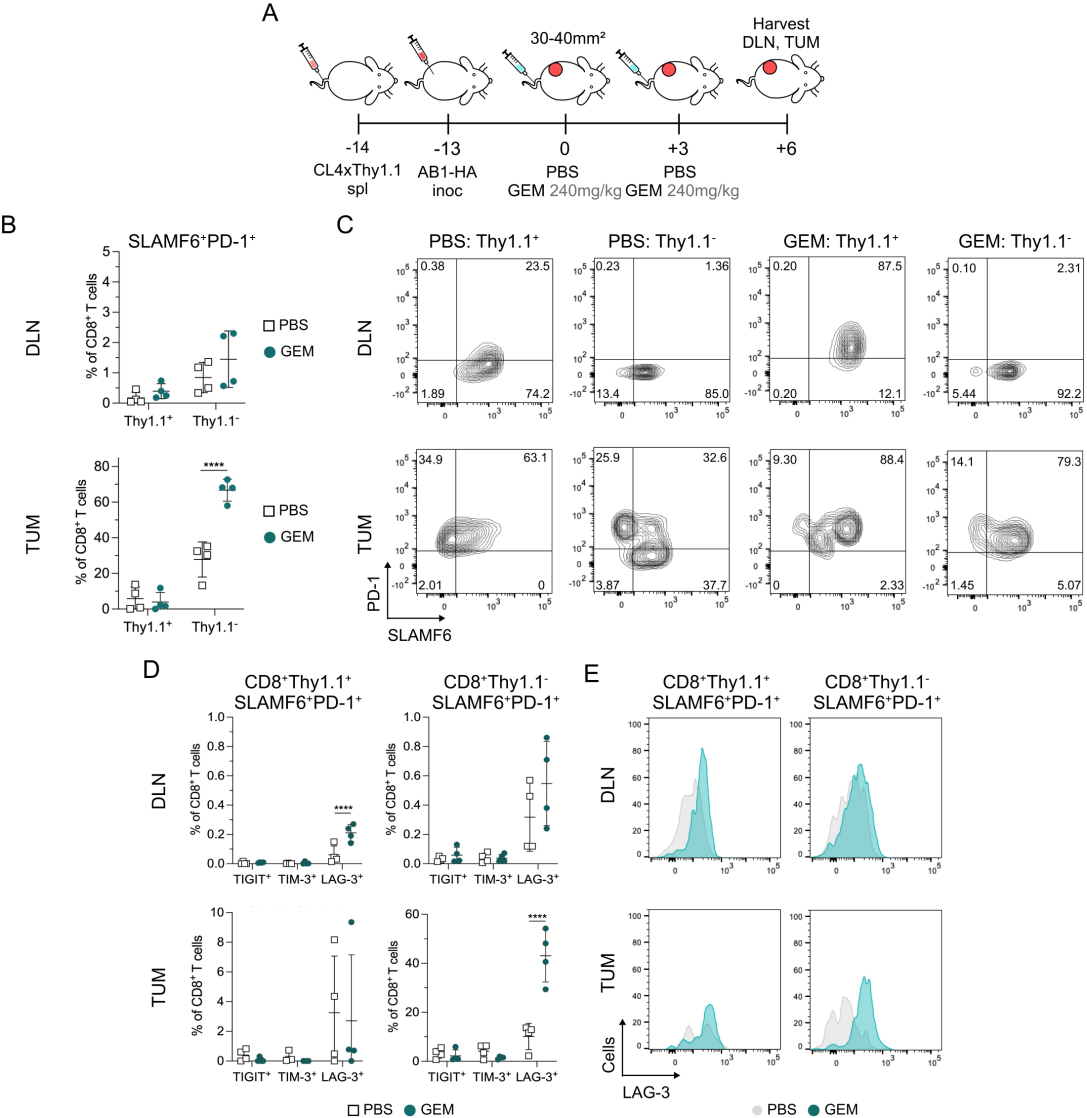
Chemotherapy induced increase in SLAMF6^+^PD-1^+^ CD8^+^ T_PEX_ is not restricted to tumor antigen-specific CD8^+^ T cells. (**A**) Experiment timeline. BALB/c recipients received CL4xThy1.1 splenocytes 1-day prior to AB1-HA tumor inoculation. Mice were treated with two doses of either PBS or GEM when tumors reached 30–40 mm^2^ in size. Tumors (TUM) and tumor draining lymph nodes (DLN) were harvested for flow cytometry 3 days after the second dose of chemotherapy (+6). (**B**) Dot plots showing the percentage of Thy1.1^+^ or Thy1.1^−^ SLAMF6^+^PD-1^+^ T_PEX_ out of CD8^+^ T cells in PBS and GEM treated DLNs (top) and TUM (bottom). (**C**) Representative flow cytometry plots of SLAMF6 and PD-1 expression on CD8^+^Thy1.1^+^ and CD8^+^Thy1.1^−^ T cells in DLNs (top) and TUM (bottom). (**D**) Dot plots presenting the frequency of TIGIT, TIM-3 and LAG-3 expression on CD8^+^Thy1.1^+^ (left) and CD8^+^Thy1.1^−^ (right) T cells in DLNs (top) and TUM (bottom). (**E**) Representative histograms of LAG-3 expression on CD8^+^Thy1.1^+^ (left) and CD8^+^Thy1.1^−^ (right) T_PEX_ in DLNs and TUM. Data represented as mean±SD. Two-way analysis of variance with Tukey’s multiple comparisons test was used to compare between treatment groups and cell types. Sample sizes n=4 per treatment group. *p<0.05, **p<0.01, ****p≤0.0001. GEM, gemcitabine; LAG-3, lymphocyte activating gene-3; PBS, phosphate-buffered saline; PD-1, programmed cell death protein 1; SALMF6, SLAM family member 6; TIGIT, T-cell immunoreceptor with immunoglobulin and ITIM domain; TIM-3, T-cell immunoglobulin and mucin-domain containing-3; T_PEX_, progenitor exhausted CD8^+^ T cells.

To determine whether sequential doses of GEM increased tumor-antigen specific LAG3^+^ CD8^+^ T_PEX_ in vivo, we transferred CD8^+^ TCR transgenic cells from CL4xThy1.1 mice into recipient BALB/c mice prior to inoculation of AB1-HA and tracked tumor-antigen (HA) specific CD8^+^ T cells by allelic marker Thy1.1.^31^ We characterized CD8^+^ T_PEX_ after two doses of GEM, because we observed the greatest differences in LAG-3 expression on CD8^+^ T_PEX_ at this time point (day+6; figure 3A). The overall frequencies of tumor-antigen specific Thy1.1^+^CD8^+^ T cells between PBS and GEM-treated animals were similar in DLNs and tumors(online supplemental figure S4E). Two doses of GEM significantly increased the frequency of endogenous tumor infiltrating Thy1.1^−^ CD8^+^ T_PEX_ (27.5±9.89% in PBS to 66.7±6.21% in GEM; p≤0.0001), but not frequencies in the HA-specific Thy1.1^+^ CD8^+^ T_PEX_ (figure 3B,C). GEM increased the frequency of LAG-3^+^ Thy1.1^+^ CD8^+^ T_PEX_ in DLNs (p≤0.0001; figure 3D,E). Expression of LAG-3 significantly increased on Thy1.1^−^ CD8^+^ T_PEX_ (p≤0.0001) but not Thy1.1^+^ CD8^+^ T_PEX_ (p=0.98) in tumors after GEM (figure 3D,E, online supplemental figure S4F). This indicated that the increased LAG-3 expression induced by GEM in vivo was restricted to tumor-antigen specific CD8^+^ T_PEX_ in DLNs but not in tumors.

### TIGIT, PD-1 and LAG-3 upregulated on intratumoral regulatory CD4^+^ T cells and conventional CD4^+^ T cells after multiple doses of anti-metabolite chemotherapy

**Figure 4:**
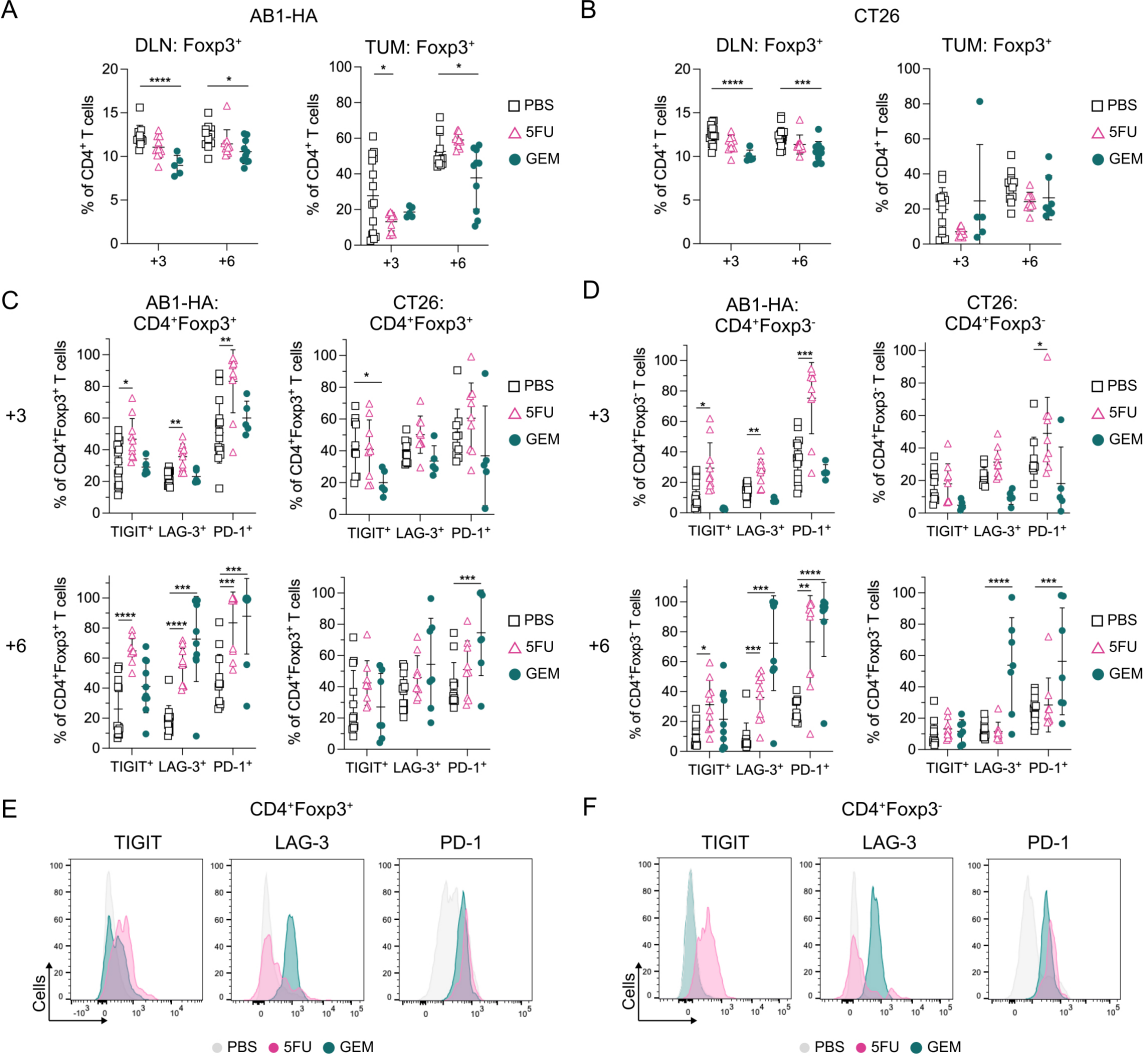
PD-1, LAG-3and TIGIT upregulate on intratumoral Tregs and Tconv after sequential chemotherapy. (**A–B**) Dot plots showing frequencies of CD4^+^Foxp3^+^ T cells in DLNs and tumors in AB1-HA (**A**) and CT26 (**B**) tumor bearing mice after one (+3) or two (+6) doses of chemotherapy. (**C–D**) Dot plots showing percentages of TIGIT, LAG-3 and PD-1 on CD4^+^Foxp3^+^ Tregs (**C**) and CD4^+^Foxp3^−^ Tconv (**D**) in AB1-HA and CT26 tumors after one (+3; top) or two (+6; bottom) doses of chemotherapy. (**E–F**) Representative histograms comparing TIGIT, LAG-3 and PD-1 on Tregs (**E**) and Tconv (**F**) after two doses of PBS, 5FU or GEM chemotherapy in AB1-HA tumors. Data represented as mean±SD. Ordinary two-way analysis of variance with Tukey’s multiple comparisons test was used to compare between treatment groups and time points. Sample sizes for flow cytometry experiments were n=5–10 per treatment group. *p<0.05, **p<0.01, ***p<0.001,****p≤0.0001. DLN, draining lymph nodes; GEM, gemcitabine; LAG-3, lymphocyte activating gene-3; PBS, phosphate-buffered saline; PD-1, programmed cell death protein 1; Tconv, conventional CD4^+^ T cells; TIGIT, T-cell immunoreceptor with immunoglobulin and ITIM domain; TIM-3, T-cell immunoglobulin and mucin-domain containing-3; Treg, regulatory CD4^+^ T cells; TUM, tumors; 5-FU, 5-fluorouracil.

As LAG-3 was increased on CD8^+^ T_PEX_ after chemotherapy, we examined whether inhibitory receptor expression was altered on intratumoral CD4^+^ regulatory (Tregs; CD4^+^Foxp3^+^) and conventional (Tconv; CD4^+^Foxp3^−^) T cells, as chemotherapy is reported to alter the activation and differentiation status of these cells.^36 37^ In both AB1-HA and CT26 models (figure 1B), one (day+3) or two (day+6) doses of GEM significantly decreased the overall proportion of Tregs in DLNs compared with PBS controls (figure 4A.B). Two doses of GEM significantly decreased Treg proportions in AB1-HA (p=0.03) but not CT26 tumors.

5FU treated AB1-HA tumors displayed an increase in proportions of Tregs (CD4^+^Foxp3^+^) expressing TIGIT, LAG-3, and PD-1 compared with PBS controls at both time points, which was not found in CT26 (figure 4C). Two doses of GEM significantly increased the frequency of LAG-3^+^ Tregs in AB1-HA (p=0.0004) but not in CT26 tumors. The proportion of PD-1^+^ Tregs significantly increased after two doses of GEM in both AB1-HA (p=0.0008) and CT26 (p=0.001) tumors(figure 4C).

For Tconv (CD4^+^Foxp3^−^), one dose of 5FU (day+3) increased the expression of PD-1 (AB1-HA: p=0.0008; CT26: p=0.01), and TIGIT (AB1-HA: p=0.02; CT26: p=0.08) compared with PBS controls (figure 4D). The proportion of PD-1^+^ or LAG-3^+^ Tconv was significantly increased after two doses of 5FU (LAG-3: p=0.0008; PD-1: p=0.002), or GEM (LAG-3: p=0.0003; PD-1: p≤0.0001) in the AB1-HA model but only after two doses of GEM in the CT26 model (LAG-3: p≤0.0001; PD-1: p=0.0002). In addition, the frequency of TIGIT^+^ Tconv significantly increased after two doses of 5FU in AB1-HA (p=0.011), but not CT26 tumors (figure 4D). TIM-3 or CTLA-4 expression on Tregs and Tconv were not altered following one or two doses of chemotherapy (online supplemental figure S5A,B).

We defined co-expression of all five inhibitory checkpoint receptors on Tregs and Tconv after chemotherapy in both tumor models (online supplemental figure S5C–F). LAG-3 and PD-1 were the predominant inhibitory receptors co-expressed on Tregs and Tconv that significantly increased after one or two doses of chemotherapy across both models (figure 4E,F, online supplemental figure S5C–F). For both PBS and chemotherapy treated tumors, there were greater numbers of LAG-3^+^ Tconv than Tregs (online supplemental figure S6A,B). LAG-3 expression also increased on Tregs and Tconv after two doses of GEM in DLNs in CT26 but not in AB1-HA (online supplemental figure S6C,D). Multiple doses of anti-metabolite chemotherapy increased inhibitory receptor expression on tumor infiltrating CD4^+^ T cells.

### Increase in LAG-3 expressing TILs is restricted to anti-metabolite chemotherapy

To assess whether the changes in frequencies of LAG-3 expressing TILs extended to other chemotherapies such as CP and CTX, we characterized LAG-3 expression on CD8^+^ T_PEX_, Tregs and Tconv in AB1-HA and CT26. Although CD8^+^ T_PEX_ increased in CTX treated tumors (figure 1G), the frequency of LAG-3^+^ CD8^+^ T_PEX_ did not significantly increase compared with PBS controls in both models (online supplemental figure S7A). CTX treated AB1-HA tumors had significantly lower frequencies of Tregs (p=0.002; online supplemental figure S7B). The frequency of LAG-3^+^ Tregs or Tconv was similar between CTX, CP and PBS tumors in both AB1-HA and CT26 models (online supplemental figure S7C,D). CP significantly increased the proportions of intratumoral SLAMF6^−^PD-1^+^CD8^+^ T cells in CT26 (p=0.0019) (online supplemental figure S7E), which were expressing TIM-3 (online supplemental figure S7F), suggesting that other classes of chemotherapy augments TIL subsets in a different manner.

We also evaluated GEM and 5FU chemotherapies in additional tumor models on a different genetic background (C57BL/6), namely AE17 mesothelioma and MC38 colon cancer (online supplemental figure S7G). In both models, proportions of LAG-3^+^CD8^+^ T_PEX_ were similar between PBS and chemotherapy treated tumors (online supplemental figure S7H, I). Two doses of 5FU significantly increased LAG-3^+^ Tregs compared with PBS controls in both models (AE17: p=0.03; MC38: p=0.01; online supplemental figure S7J), and increased intratumoral LAG-3^+^ Tconv in MC38 (p=0.01) (online supplemental figure S7K). In additional models, LAG-3 expression on CD4^+^ TILs are altered after anti-metabolite chemotherapy.

### Combination of anti-PD-1 and anti-LAG-3 ICB after anti-metabolite chemotherapy generates robust anti-tumor responses in multiple tumor models

**Figure 5:**
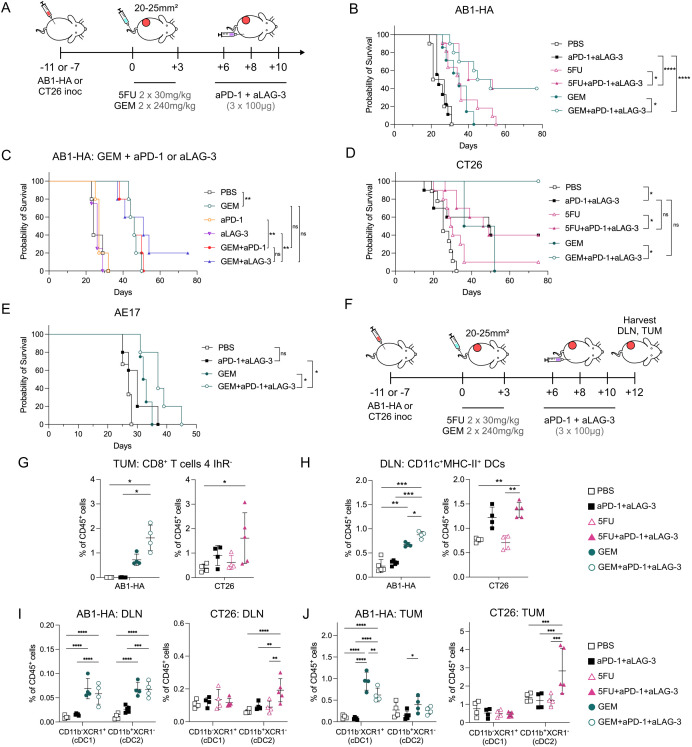
aPD-1 and aLAG-3 ICB with sequential chemotherapy improves the antitumor immune response. (**A**) Experiment timeline. AB1-HA or CT26 tumor bearing animals were treated with 5FU, GEM or PBS when tumors reached 20–25 mm^2^ in size. aPD-1 and aLAG-3 ICB were administered 3 days after the second dose of chemotherapy (+6), the same time point as T-cell phenotyping experiments. (**B**) Survival curves of AB1-HA tumor bearing animals treated with GEM or 5FU chemotherapy in combination with aPD-1 and aLAG-3 ICB. Sample sizes were n=10 per treatment group from two pooled experiments. (**C**) Survival curves of AB1-HA tumor-bearing animals treated with aPD-1 or aLAG-3 in combination with GEM (n=5 per treatment group, one experiment). (**D**) Survival curves of CT26 tumor bearing animals treated with GEM or 5FU chemotherapy in combination with aPD-1 and aLAG3 ICB. Sample sizes were n=10 per treatment group from two pooled experiments except GEM (n=2) and GEM+aPD-1+aLAG-3 (n=4). (**E**) Survival curves of AE17 tumor bearing animals treated with GEM chemotherapy in combination with aPD-1 and aLAG-3 (n=5 per treatment group, one experiment). (**F**) Experiment timeline. DLN and tumors were harvested 2 days after the full chemo-immunotherapy schedule. (**G**) Dot plots representing frequencies of TIGIT^−^PD-1^−^LAG-3^−^CTLA-4^−^ CD8^+^ T cells (4 IhR^−^) in AB1-HA (left) or CT26 (right) tumors. (**H**) Dot plots displaying frequencies of CD11c^+^MHC-II^+^ dendritic cells in DLNs from AB1-HA (left) and CT26 (right). (**I–J**) Dot plots showing proportion of conventional dendritic cells (CD11c^+^MHC-II^+^): cDC1 (CD11b^−^XCR1^+^) and cDC2 (CD11b^+^XCR1^−^) in AB1-HA (left) and CT26 (right) tumors (**I**) and DLNs (**J**). Data represented as mean±SD. Survival experiments: Mantel-Cox survival test. Flow cytometry experiments: n=4–5/group. Ordinary two-way analysis of variance with Tukey’s multiple comparisons test was used to compare between treatment groups. *p<0.05, **p<0.01, ***p<0.001,****p≤0.0001. aLAG-3, anti-LAG-3; aPD-1, anti-PD-1; cDC, conventional dendritic cell; DC, dendritic cell; DLN, draining lymph nodes; GEM, gemcitabine; ICB, immune checkpoint blockade; TIGIT: T cell immunoreceptor with immunoglobulin and ITIM domain; LAG-3, lymphocyte activating gene-3; CTLA-4: cytotoxic T lymphocyte antigen 4; MHC, major histocompatibility complex; PBS, phosphate-buffered saline; PD-1, programmed cell death protein 1; TUM, tumors.

As LAG-3 expression increased on TILs including CD8^+^ T_PEX_ in the tumors after anti-metabolite chemotherapy, we sought to determine if adding anti-(a)LAG-3 with anti-(a)PD-1 ICB would improve anti-tumor immunity after chemotherapy. Administering aLAG-3 and aPD-1 3 days after two doses of either 5FU or GEM (day+6) significantly increased median survival compared with each agent alone in AB1-HA (figure 5A,B). Complete tumor regression occurred in 40% of chemotherapy+aLAG-3+aPD-1 ICB-treated animals compared with 0% in those treated with chemotherapy (5FU: p=0.02; GEM: p=0.01) or aPD-1/a-LAG-3 alone (GEM: p≤0.0001; 5FU: p≤0.0001). To ensure this effect was not due to greater likelihood of complete tumor regression by administering ICB to mice with a small tumor burden, we administered aPD-1+aLAG-3 ICB to 9–20 mm^2^ AB1-HA tumors, which is the approximate tumor size seen after two doses of either 5FU or GEM. We found that aPD-1+aLAG-3 ICB had no significant effect on growth compared with PBS controls (online supplemental figure S8A), indicating that the combination of 5FU or GEM with aPD-1+aLAG-3 ICB was required to generate a robust anti-tumor response for AB1-HA.

As GEM also increased the proportion of CD8^+^ T_PEX_ that expressed PD-1, but not LAG-3 (figure 2C,D), we wanted to determine if single agent ICB would induce similar results to multi-agent ICB in combination with chemotherapy. There was no significant difference in survival between GEM alone, or GEM with single agent aPD-1 (p=0.56) or aLAG-3 ICB (p=0.21; figure 5C), indicating that combination aPD-1 and aLAG-3 ICB was required to generate the robust anti-tumor responses observed.

In CT26, both 5FU and GEM with aPD-1+aLAG-3 ICB significantly improved survival compared with respective chemotherapy alone (5FU: p=0.02; GEM: p=0.01), however combined aPD-1 and aLAG-3 was just as effective, producing 40% complete cures, the same as in combination with 5FU (figure 5D). Lastly, we sought to determine if this chemo-immunotherapy combination, with the same scheduling, could be effective in AE17 mesothelioma. We found that GEM+aPD-1+aLAG-3 ICB significantly improved survival compared with GEM (p=0.04) or aPD-1+aLAG-3 ICB alone (p=0.02; figure 5E). These data demonstrate that aPD-1+aLAG-3 ICB after multiple doses of anti-metabolite chemotherapy successfully improves the anti-tumor immune response.

To understand how GEM and 5FU combined with aPD-1+aLAG-3 improved anti-tumor immunity, we characterized immune cells from tumors and DLNs 2 days after the completion of the treatment schedule (figure 5F). As combination therapy could augment different cell types to improve anti-tumor immunity, we characterized CD8^+^, Tconv (CD4^+^Foxp3−), Tregs (CD4^+^Foxp3^+^), DCs (CD11c^+^MHC-II^+^), monocytic (CD11b^+^Ly6C^+/−^F480^+/−^CD64^+/−^) and neutrophilic (CD11b^+^Ly6G^+^) derived myeloid cell populations (online supplemental figure S8C). We analyzed 5FU+aPD-1+aLAG-3 in CT26, and GEM+aPD-1+aLAG-3 in AB1-HA, comparing chemo-immunotherapy with respective chemotherapies, or aPD-1+aLAG-3 ICB.

There was no consistent pattern of change in frequencies of CD45^+^, CD8^+^, Tconv and Tregs resulting from combination treatment in both models (online supplemental figure S8B,C). Proportions of CD45^+^ cells were similar between treatment groups for DLNs and tumors except in AB1-HA tumors, where combination therapy or aPD-1+aLAG-3 ICB increased the proportion of CD45^+^ cells compared with PBS or GEM alone (online supplemental figure S8B). An increase in LAG-3^+^SLAMF6^+^PD-1^+^ CD8^+^ T_PEX_ frequency was only found in DLNs from GEM+aPD-1+aLAG-3 treated animals compared with PBS controls (p=0.03), but not in tumors or in the 5FU model (online supplemental figure S8D). In both models, the frequencies of tumor infiltrating CD8^+^ T cells without inhibitory receptor expression (4 IhR^−^) significantly increased in chemo-immunotherapy compared with PBS controls (AB1-HA: p=0.01; CT26: p=0.04; figure 5G). The frequency of proliferating (Ki67^+^) CD8^+^ 4IhR^−^ T cells increased in combination therapy compared with chemotherapy alone (online supplemental figure S8E), suggesting chemo-immunotherapy enhanced proliferation of less differentiated populations of CD8^+^ T cells.

As improved antigen-presentation with GEM was observed in previous experiments, we characterized DCs in both chemo-immunotherapy models. The overall frequency of DCs (CD11c^+^MHC-II^+^) significantly increased in DLNs from chemo-immunotherapy compared with ICB (AB1-HA: p=0.0001), chemotherapy (AB1-HA: p=0.01; CT26: p=0.001) or PBS (AB1-HA: p=0.0001; CT26: p=0.001) controls (figure 5H). CD11b and XCR1 expression was used to identify conventional dendritic cell (cDC) subsets. In AB1-HA tumors and DLNs, GEM+aPD-1+aLAG-3, or GEM alone significantly increased cDC1 (CD11c^+^MHC-II^+^CD11b^−^XCR1^+^) compared with ICB (p<0.0001) (figure 5I,J). In CT26 tumors and DLNs, 5FU+aPD-1+aLAG-3 significantly increased cDC2 (CD11c^+^MHC-II^+^ CD11b^+^XCR1^−^) compared with ICB (p=0.0002) or 5FU alone (p=0.0002) (figure 5I,J). The frequency of CD103^+^ cDC1 significantly increased in GEM+aPD-1+aLAG-3 DLNs compared with PBS (p=0.0016), suggesting trafficking of cDC1s from the tumor to the DLN (online supplemental figure S8F). We compared expression of activation (CD86), migration (CX3CR1) and LAG-3/PD-1 ligands (galectin 3, PD-L1, PD-L2) on DCs. Combination GEM+aPD-1+aLAG-3, and GEM alone increased the proportions of DLN CD86^+^ DCs when compared with PBS (p=0.0002) or ICB (p=0.004). Combination 5FU+aPD-1+aLAG-3 increased CD86^+^ DCs compared with PBS (p=0.004), 5FU (p=0.04), or ICB (p=0.03) alone. There were no consistent changes in other markers on DCs across both models (online supplemental figure S8G). Lastly, we analyzed activation and inhibitory markers on macrophages, monocytic and neutrophilic myeloid cells and likewise did not observe differences that were present in both tumor models (online supplemental figure S8H–J). In the models assessed, aPD-1+aLAG-3 ICB in combination with anti-metabolite chemotherapy improves anti-tumor immunity by increasing CD8^+^ T-cell proliferation and DCs proportions in tumors and DLNs.

## Discussion

Here we analyzed CD4^+^ and CD8^+^ T cells following multiple doses of anti-metabolite chemotherapies in two murine tumor models to identify ICB targets to combine with chemotherapy. We found two doses of anti-metabolite chemotherapies increased frequencies of intratumoral CD8^+^ T_PEX_ and enhanced expression of LAG-3 on CD8^+^ T_PEX_, Tconv and Tregs. Anti-LAG-3 and anti-PD-1 ICB in combination with 5FU or GEM chemotherapy was an effective chemoimmunotherapy combination in mesothelioma and colon cancer preclinical models.

5FU and GEM have been reported to enhance CD8^+^ T-cell activation in tumors.^8 38 39^ Our study adds to this by demonstrating that sequential anti-metabolite chemotherapy and CTX increased CD8^+^ T_PEX_ in tumors, an important subset of T cells that mediates anti-tumor responses in the context of ICB. By comparing the transcriptomes of matched pre-chemotherapy and on-chemotherapy (doxorubicin and CTX) breast cancer biopsies, we likewise found enrichment of CD8^+^ T_PEX_ signatures. Guan and colleagues reported increased intratumoral CD8^+^ T_PEX_ after FOLFOX chemotherapy (5FU, oxaliplatin) in preclinical colon cancer models.^40^ Higher TCF1 expression suggesting increased CD8^+^ T_PEX_ proportions was also reported after CTX and vinorelbine, when combined with anti-PD-1.^41^ The effects of single agent or combination chemotherapy, on CD8^+^ T_PEX_ in humans are still not well understood because CD8^+^ T_PEX_ are mostly studied in the context of ICB, and not in chemotherapies alone. Yan and colleagues reported that carboplatin and paclitaxel chemotherapies increased CX3CR1^+^CD8^+^ T cells in patients with melanoma that subsequently responded to anti-PD-1 ICB. These cells could have a progenitor exhaustive phenotype.^42^ Further transcriptional, epigenetic and molecular characterization of CD8^+^ T_PEX_ with markers such as CD69, Tox and Blimp1 will enable us to understand how different chemotherapies alters CD8^+^ T_PEX_ in a way that favors ICB treatment.

5FU or GEM increased LAG-3 expression on intratumoral CD8^+^ T_PEX_, Tregs and Tconv in both models. LAG-3 marks activated CD4^+^ and CD8^+^ T cells, which is consistent with increased activation of these immune cell subsets by chemotherapy.^13^ As a negative regulator, LAG-3 inhibits TCR downstream signaling, thereby negatively modulating T-cell expansion and cycling.^43^ We speculate that increased immunogenic cell death^44 45^ activates intratumoral DCs^10 11^ which in turn activates T cells and increases LAG-3 expression. Chemotherapy could also selectively deplete subsets of CD8^+^ TILs,^42^ but the direct effects of chemotherapy on SLAMF6/PD-1 expressing CD8^+^ TILs are unclear. Interestingly, GEM enhanced the frequency of tumor-antigen (HA) specific LAG-3^+^ CD8^+^ T_PEX_ in the DLN, and non-HA specific LAG-3^+^ CD8^+^ T_PEX_ in the tumors. LAG-3 could be a marker for endogenous CD8^+^ T cells in the tumors that are specific for tumor antigens other than HA. Alternatively, GEM could be increasing non tumor -antigen specific LAG-3^+^ CD8^+^ T_PEX_ in the tumor. Both tumor specific and bystander CD8^+^ T cells have been shown to exert anti-tumor activity within tumors.^46 47^ The antigen-specificity of LAG-3^+^ CD8^+^ T_PEX_ in the these models remains to be elucidated.

We demonstrate that expression of inhibitory checkpoint receptors on T cells after anti-metabolite chemotherapy provides rationale to select ICB targets. In this case, aLAG-3 and aPD-1 ICB following anti-metabolite chemotherapy significantly improved survival in two mesothelioma models, achieving complete tumor regression in most animals compared with monotherapy controls in one mesothelioma model. In CT26, aLAG-3 and aPD-1 ICB alone achieved tumor regression similar to combination chemotherapy with aLAG-3 and aPD-1 ICB. This is in line with our results showing that anti-metabolite chemotherapy increased LAG-3 expression on CD8^+^ T_PEX_ to a lesser degree in CT26 compared with AB1-HA. aLAG-3 and aPD-1 have been shown to be effective in multiple preclinical models,^48^,^50^ and to our knowledge, this is the first demonstration of an effective combination of these ICB therapies with multiple doses of chemotherapy. We speculate that aLAG-3 and aPD-1 ICB in combination with anti-metabolite chemotherapy increases T-cell proliferation by improving DCs numbers and function in tumors and their corresponding DLNs.^49 51^ Serial tumor biopsies before and after the first cycles of chemotherapy could provide guidance of ICB selection in the clinic.

There is limited clinical data on aLAG-3 and aPD-1 ICB in combination with chemotherapy. The first trial with aLAG-3 ICB was in combination with paclitaxel chemotherapy for patients with breast cancer in which the combination enhanced clinical outcomes and reduced toxicities.^52^ Relatlimab was the first aLAG-3 ICB to be developed and is currently being evaluated across multiple cancer types. In patients with melanoma, relatlimab and nivolumab (anti-PD-1) significantly improved overall survival compared with nivolumab alone.^53^ Various clinical trials are underway to assess to benefit of combination chemotherapy and relatlimab with/without nivolumab including gastric cancer (NCT04062656) and non-small cell lung cancer (NCT04623775). Our preclinical study provides a rationale to study aLAG-3 and aPD-1 ICB in combination with anti-metabolite chemotherapy for cancers such as malignant mesothelioma.

This study was limited by the number of chemotherapies tested, and we speculate that other chemotherapies could result in different changes in inhibitory receptor expression on TILs. As an example, we observed increased TIM-3, instead of LAG-3 on CD8^+^ TILs in CP treated tumors. There were also minor discrepancies as not all mouse models displayed an increase in LAG-3 expressing CD8^+^ T_PEX_ after chemotherapy. This could be attributed to heterogeneity in TIL differentiation state, and frequencies of T_PEX_ between tumor models found at baseline. Lastly, we focused on a single time point after treatment initiation, further mechanistic understanding of chemotherapies in combination with immunotherapy requires careful analysis of TILs and DLNs over time.

In conclusion, our study underscores the need to understand how chemotherapy influences immune cells in the tumor microenvironment to improve sensitivity to ICB. Serial monitoring of the tumor immune milieu throughout chemotherapy treatment, where possible, could provide individualized selection of ICB therapies. These data may have a significant impact on the future development of clinical trials to select efficacious chemotherapy and ICB combinations for patients with cancer.

## supplementary material

10.1136/jitc-2023-008568 online supplemental file 1

## Data Availability

All data relevant to the study are included in the article or uploaded as supplementary information.
