# Supplementary material for: Anti-metabolite chemotherapy increases LAG-3 expressing tumor-infiltrating lymphocytes which can be targeted by combination immune checkpoint blockade
Source: J Immunother Cancer. 2024 Sep 28;12(9):e008568. doi: 10.1136/jitc-2023-008568 (PMC11440230; doi:10.1136/jitc-2023-008568)
Supplement: online supplemental file 1 [file jitc-12-9-s001.docx]

**Supplementary Material**

***Anti-metabolite chemotherapy increases LAG-3 expressing tumour-infiltrating lymphocytes which can be targeted by combination immune checkpoint blockade***

**Table S1. Flow cytometry antibodies**

| Fluorochrome | Antigen | Dilution | Clone | Supplier | Catalog No. |
| --- | --- | --- | --- | --- | --- |
| BUV395 | CD3 | 1:250 | 145-2C11 | BD | 563565 |
| Zombie UV | FVD | 1:4000 |  | Biolegend | 423107 |
| BV510 | CD8a | 1:250 | 53-6.7 | BD | 563068 |
| BV605 | TIM3 | 1:100 | RMT3-23 | Biolegend | 119721 |
| BV711 | TIGIT | 1:100 | 1G9 | BD | 744214 |
| FITC | Foxp3 | 1:500 | FJK-16s | eBioscience | 11-5773-82 |
| AF488 | Thy1.1 (CD90) | 1:200 | OX-7 | Biolegend | 202505 |
| AF488 | TCF1/7 | 1:500 | CD63D9 | Cell Signalling | 644S |
| PerCP-Cy5.5 | CTLA-4 | 1:100 | UC10-4B9 | Biolegend | 106315 |
| PE | SLAMF6 | 1:1000 | 13G3 | BD | 561540 |
| PE-Dazzle | Ki67 | 1:500 | 16A8 | Biolegend | 652427 |
| PE-Cy7 | LAG-3 | 1:100 | C9B7W | eBioscience | 25-2231-80 |
| APC | PD-1 | 1:500 | RMP1-30 | Biolegend | 109111 |
| APC-Cy7 | CD45 | 1:1000 | 30-F11 | BD | 561037 |
| AF488 | CD107a | 1:250 | 1D4B | Biolegend | 121606 |
| FITC | IFNg | 1:250 | XMG1.2 | eBioscience | 11-7311-41 |
| APC | CD137 | 1:100 | 17B5 | eBioscience | 17-1371-82 |
| BV605 | Tbet | 1:100 | 4B10 | Biolegend | 644817 |
| Pacific Blue | GzmB | 1:100 | GB11 | Biolegend | 515407 |
| Viability | FVS780R | 1:20000 |  | Invitrogen | 65086514 |
| FITC | Ly6C | 1:200 | HK1.4 | Biolegend | 128006 |
| BB700 | F4/80 | 1:400 | T45-2342 | BD | 746070 |
| RB780 | PDL1 | 1:200 | MIH5 | BD | 755394 |
| AF647 | CD86 | 1:200 | GL-1 | BioLegend | 105020 |
| AF700 | Ly6G | 1:400 | 1A8 | BD | 561236 |
| BUV395 | XCR1 | 1:200 | ZET | Biolegend | 148236 |
| BUV496 | CD11b | 1:500 | M1/70 | BD | 749864 |
| BUV563 | PDL2 | 1:200 | TY25 | BD | 741431 |
| BV421 | CD103 | 1:100 | M290 | BD | 562771 |
| BV510 | MHCII | 1:200 | M5/114.15.2 | BD | 742893 |
| BUV570 | CD45 | 1:200 | 30-F11 | Biolegend | 103136 |
| BV605 | CD11c | 1:200 | N418 | BioLegend | 117334 |
| BV650 | CCR2 | 1:200 | 475301 | BD | 747968 |
| BV711 | CD64 | 1:200 | X54-5/7.1 | BioLegend | 139311 |
| BV786 | CX3CR1 | 1:200 | SA011F11 | Biolegend | 149029 |
| PE-Cy7 | Galectin3 | 1:500 | M3/38 | Biolegend | 125418 |

**Table S2. Summary of immune cell characterisation after chemotherapy and aPD-1+aLAG-3 ICB**

| Cell type | Sample | AB1-HA: GEM+ICB | | CT26: 5FU+ICB | |
| --- | --- | --- | --- | --- | --- |
|  |  | Change | P value | Change | P value |
| CD8^+^ T cells | DLN | None |  | None |  |
|  | TUM | None |  | None |  |
| CD8^+^ LAG-3^+^ SLAMF6^+^PD-1^+^ | DLN | Increase | PBS: p = 0.03 | None |  |
|  | TUM | None |  | None |  |
| CD8^+^ SLAMF6^+^PD-1^+^ | DLN | None |  | None |  |
|  | TUM | None |  | None |  |
| CD8^+^ SLAMF6^-^PD-1^+^ | DLN | None |  | None |  |
|  | TUM | None |  | Increase | PBS: p = 0.04  ICB: p = 0.01  5FU: p = 0.001 |
| CD8^+^ SLAMF6^+^PD-1^-^ | DLN | None |  | Decrease | PBS: p < 0.0001  ICB: p = 0.02  5FU: p = 0.0006 |
|  | TUM | Increase | PBS: p = 0.02 | None |  |
| CD8^+^ 4IhR^-^ | DLN | None |  | None |  |
|  | TUM | Increase | PBS: p = 0.02  ICB: p = 0.02 | Increase | PBS: p = 0.04 |
| CD8^+^ Ki67^+^ 4IhR^-^ | DLN | None |  | None |  |
|  | TUM | Increase | PBS: p = 0.02  ICB: p = 0.02 | Increase | PBS: p = 0.03  ICB: p = 0.02 |
| CD4^+^Foxp3^-^ Tconv | DLN | None |  | Decrease | ICB: p = 0.03 |
|  | TUM | Decrease | PBS: p = 0.03 | None |  |
| CD4^+^Foxp3^+^ Tregs | DLN | Increase | PBS: p = 0.003 | None |  |
|  | TUM | Increase | PBS: p = 0.004  GEM: p = 0.03 | None |  |
| CD11c^+^MHC-II^+^ DCs | DLN | Increase | PBS: p = 0.0003  ICB: p = 0.0001  GEM: p = 0.01 | Increase | PBS: p = 0.001  5FU: p = 0.001 |
|  | TUM | Increase | PBS: p = 0.02  ICB: p < 0.0001  GEM: p = 0.02 | None |  |
| CD11c^+^MHC-II^+^ CD11b^-^XCR1^+^ (cDC1) | DLN | Increase | PBS: p < 0.0001  ICB: p < 0.0001 | None |  |
|  | TUM | Increase | PBS: p < 0.0001  ICB: p < 0.0001  GEM: p = 0.002 | None |  |
| CD11c^+^MHC-II^+^ CD11b^+^XCR1^-^ (cDC2) | DLN | Increase | PBS: p < 0.0001  ICB: p =0.0001 | Increase | PBS: p < 0.0001  ICB: p = 0.002  5FU: p = 0.001 |
|  | TUM | No change |  | Increase | PBS: p = 0.0006  ICB: p = 0.0002  5FU: p = 0.0002 |
| CD103^+^ cDC1 | DLN | Increase | PBS: p = 0.0016 | Increase | NS |
| CD11b^+^F480^+^ Ly6C^-^ Macrophages | DLN | Increase | PBS: p = 0.005  ICB: p = 0.005 | None |  |
|  | TUM | Increase | PBS: p = 0.03  ICB: p = 0.03 | None |  |
| CD11b^+^F480^lo^ Ly6C^-^ CD64^+^ Macrophages | DLN | None |  | None |  |
|  | TUM | Increase | NS | None |  |
| CD11b^+^Ly6C^hi^ Monocytes | DLN | Increase | NS | Increase | NS |
|  | TUM | Increase | PBS: p = 0.01  ICB: p = 0.008 | Increase | NS |
| CD11b^+^Ly6C^int^ Monocytes | DLN | Increase | PBS: p = 0.007  ICB: p = 0.02 | Increase | PBS: p = 0.008  5FU: p = 0007 |
|  | TUM | None |  | None |  |
| CD11b^+^Ly6G^+^ Neutrophils | DLN | None |  | None |  |
|  | TUM | Decrease | ICB: p = 0.01 | Increase | NS |

**NS: not significant, DLN: draining lymph nodes, TUM: tumours; p values derived from 2-way ANOVAs corrected for multiple comparisons; combination therapy versus respective therapies (ICB alone, chemotherapy alone or PBS)*


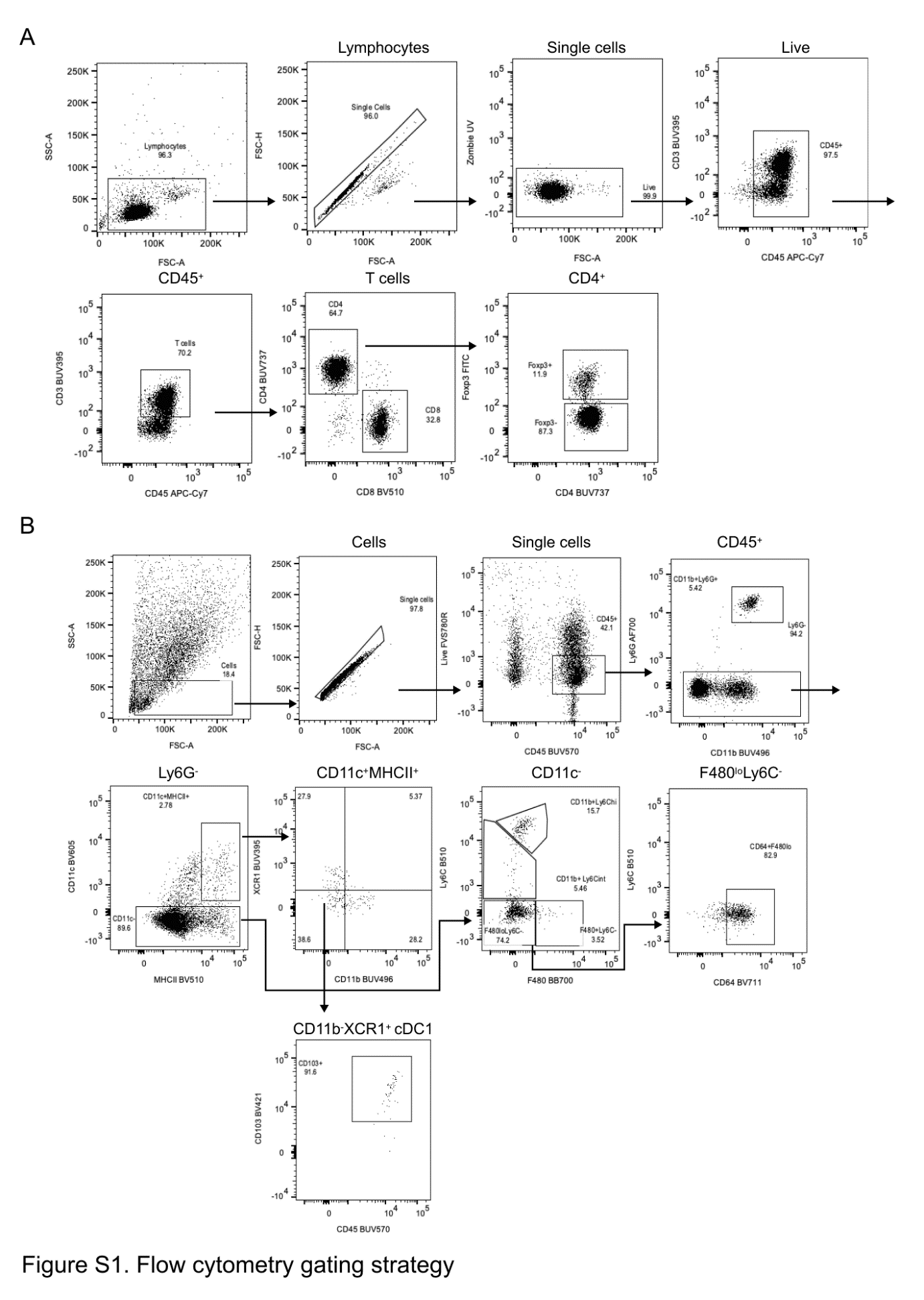


***Figure S1.* *Flow cytometry gating strategy for T cell panel (A) and myeloid panel (B)***


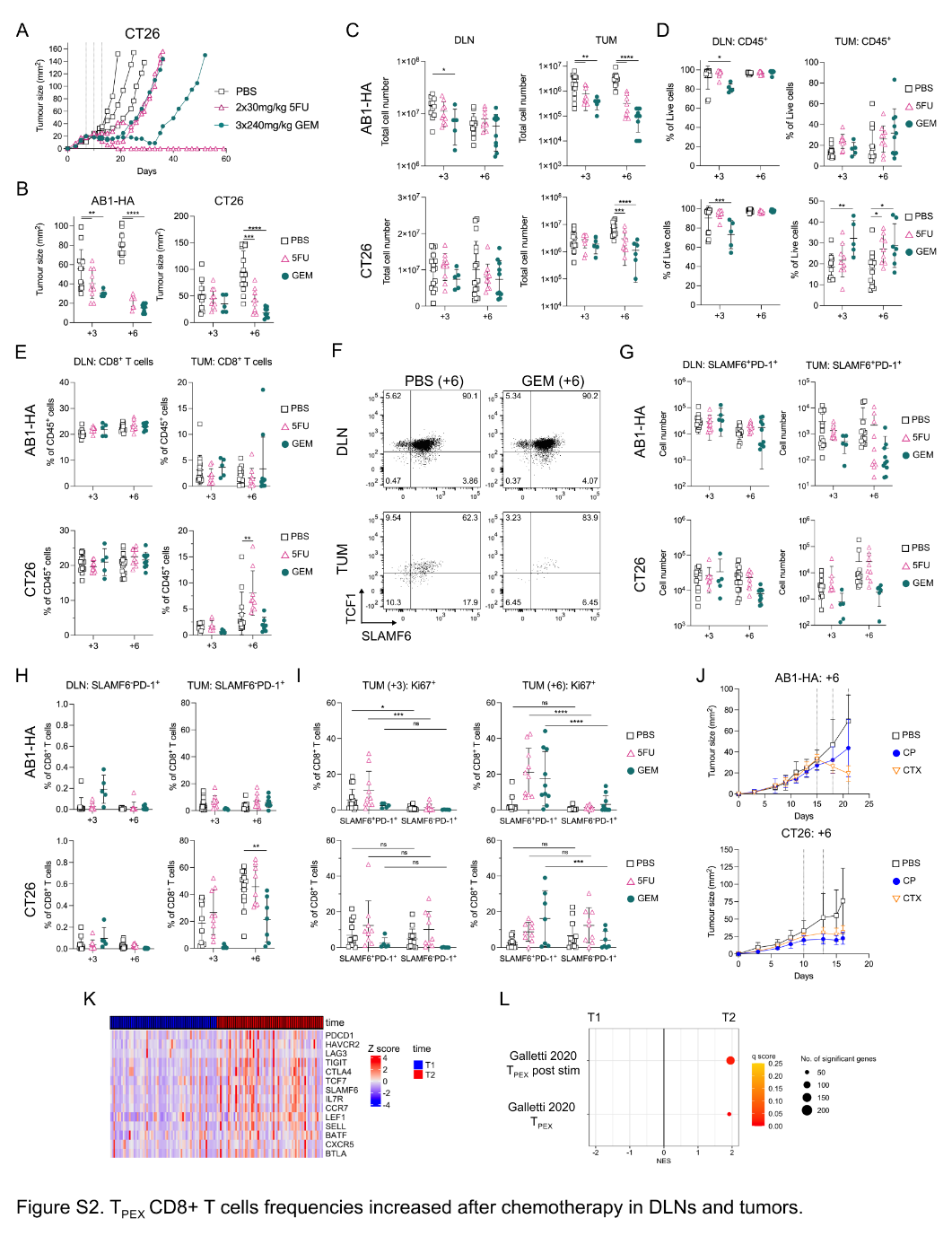


***Figure S2.* *T_PEX_ CD8^+^ T cells increased in DLNs and tumours after chemotherapy. (*A)** Representative tumour growth curves of CT26 tumour bearing mice treated with 2 doses of 30 mg/kg 5-Fluorouracil (5FU; n = 4), 3 doses of 240 mg/kg gemcitabine (GEM; n = 2) chemotherapies or PBS controls (n = 2). **(B-C)** Tumour sizes **(B)** and cell counts **(C)** of DLNs and tumours at harvest of AB1-HA and CT26 tumour bearing mice 1 (+3) or 2 (+6) doses of chemotherapy. **(D-E)** Percentages of CD45^+^ cells out of live cells **(D)**, CD8^+^ T cells out of CD45^+^ cells **(E)** in DLN and tumours at both timepoints. **(F)** Representative FACs plots of TCF1 and SLAMF6 co-expression on CD8^+^ T cells in DLNs and tumours of PBS and GEM treated mice. **(G)** Total cell numbers of SLAMF6^+^PD-1^+^ CD8^+^ T cells in DLN and tumours at both timepoints. **(H)** Proportion of SLAMF6^-^PD-1^+^ out of CD8^+^ T cells in DLNs (left) and tumours (right) after chemotherapy. **(I)** Frequency of SLAMF6^+^PD-1^+^ or SLAMF6^-^PD-1^+^ CD8^+^ T cells expressing Ki67 in tumours after one (+3: left) or two (+6: right) doses of chemotherapy. **(J)** Mean tumour growth curves of AB1-HA (left) and CT26 (right) with two doses of cisplatin (CP) or cyclophosphamide (CTX) chemotherapy. Tumours and DLNs were harvested 3 days after last dose of chemotherapy (+6) for flow cytometry. Dotted lines indicate administration of chemotherapy. **(K)** Heatmap displaying selected genes associated with CD8^+^ T cell exhaustion on pre- (T1) and on- (T2) chemotherapy treatment tumour biopsies from 68 breast cancer patients. **(L)** GSEA plot showing significant (q < 0.25) enrichment of CD8^+^ T_PEX_ gene sets on 88 T2 biopsies compared to 115 T1 unmatched biopsies collected in Park and colleagues. Data represented as mean ± SD. Two-way ANOVA with Tukey’s multiple comparisons test was used to compare between treatment groups and timepoints/cell types. Sample sizes for flow cytometry experiments was n = 5-10 per treatment group. *P < 0.05, **P < 0.01, ****P ≤ 0.0001.


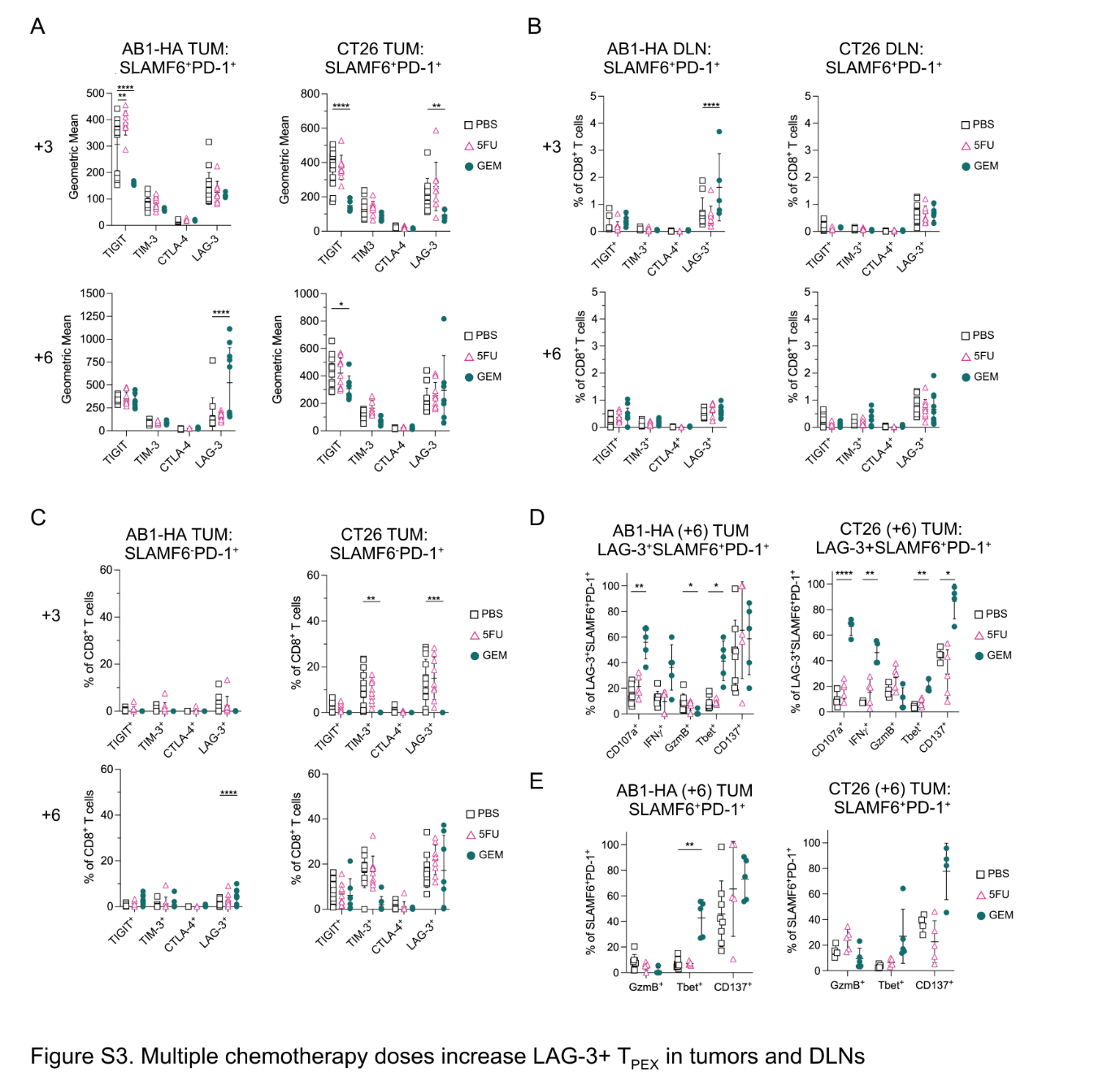


***Figure S3. LAG-3^+^ T_PEX_ in tumours and DLNs after multiple doses of anti-metabolite chemotherapy.* (A)** Dot plots displaying the geometric mean of inhibitory receptors on SLAMF6^+^PD-1^+^ CD8^+^ T cells. **(B)** Dot plots showing frequencies of inhibitory receptors on SLAMF6^+^PD-1^+^ CD8^+^ T cells in DLNs. **(C)** Dot plots representing the proportions of inhibitory receptors on SLAMF6^-^PD-1^+^ CD8^+^ T cells after 1 (+3) or 2 (+6) doses of 5FU or GEM chemotherapy in AB1-HA and CT26 tumours. **(D-E)** Dot plots displaying the frequencies of cytotoxic and effector markers on LAG-3^+^SLAMF6^+^PD-1^+^ CD8^+^ T cells **(D)** and SLAMF6^+^PD-1^+^ CD8^+^ T cells **(E)** after 2 (+6) doses of 5FU or GEM chemotherapy in AB1-HA (left) and CT26 (right) tumours. Data represented as mean ± SD. Ordinary two-way ANOVA with Tukey’s multiple comparisons test was used to compare between treatment groups and timepoints. Sample sizes for flow cytometry experiments was n = 4-10 per treatment group. *P < 0.05, **P < 0.01, ****P ≤ 0.0001.


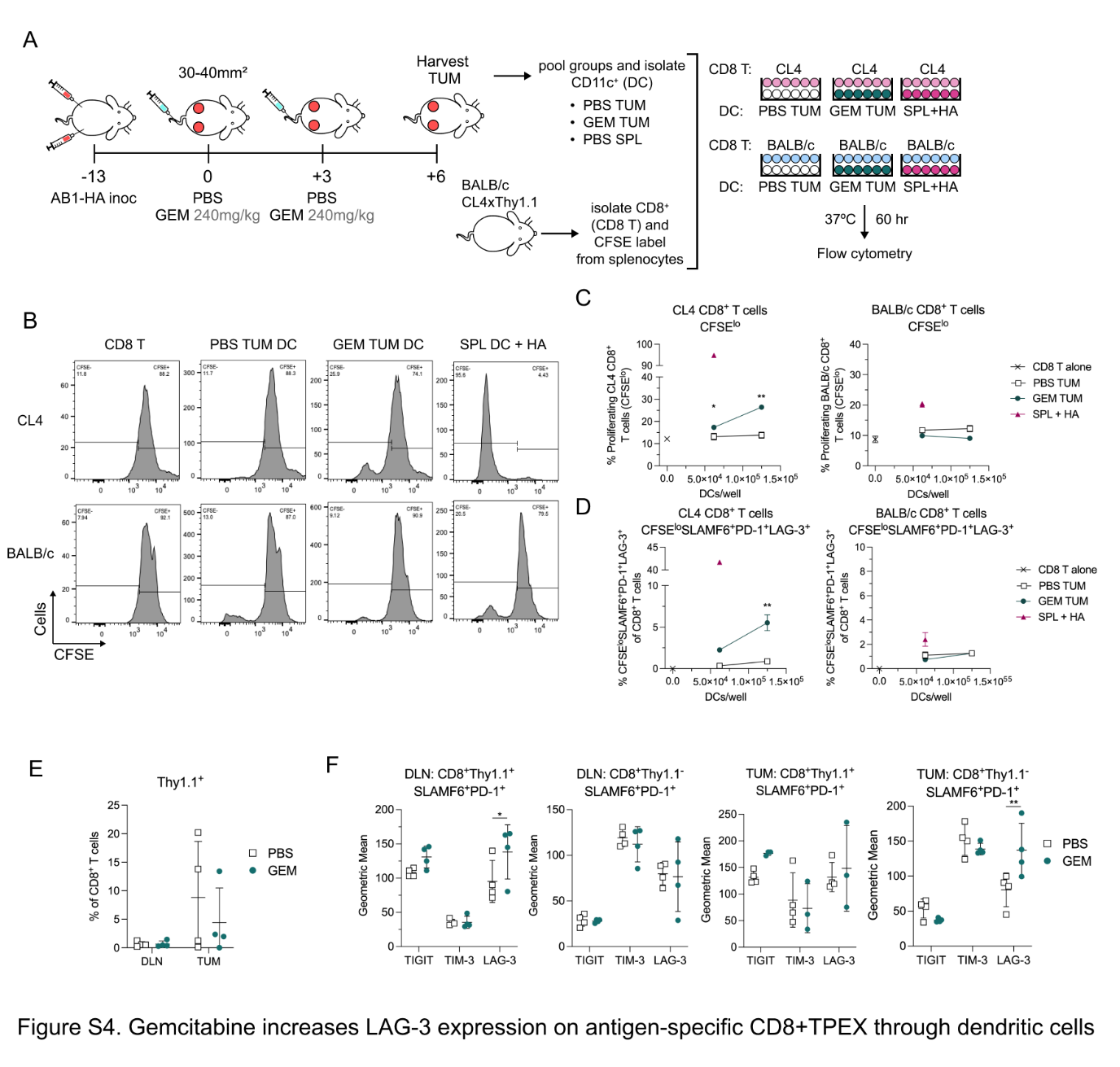


***Figure S4. Gemcitabine increases LAG-3 expression on antigen specific CD8^+^ T_PEX_ through dendritic cells.*** **(A)** Experiment timeline. AB1-HA bearing animals were treated with 2 doses of GEM or PBS when tumours reached 30-40mm^2^ in size. Tumours (TUM) and spleen (SPL) were collected after treatment and dendritic cells (DC: CD11c^+^) were isolated. CD8^+^ T cells were isolated from spleens of CL4xThy1.1 and BALB/c mice and CFSE labelled. DC-T cell co-culture was set up including dendritic cells from SPL pulsed with HA peptide as a positive control. Flow cytometry was performed 60 hours after incubation. **(B)** Histograms showing expression of CFSE on CL4 (top) and BALB/c (bottom) CD8^+^ T cells for each condition. **(C-D)** Dot plots showing the frequency of proliferating (CFSE^lo^) CD8^+^ T cells **(C)** and proliferating (CFSE^lo^) LAG3^+^SLAMF6^+^PD-1^+^ CD8^+^ T cells **(D)** from CL4xThy1.1 (left) or BALB/c (right) mice after co-culture with dendritic cells from GEM or PBS tumours. **(E)** Dot plots showing the percent of Thy1.1^+^ out of CD8^+^ T cells in PBS and GEM treated DLNs and tumours. **(F)** Dot plots presenting geometric mean of TIGIT, TIM-3 and LAG-3 on CD8^+^Thy1.1^+^ and CD8^+^Thy1.1^-^ SLAMF6^+^PD-1^+^ DLNs and tumours. Data represented as mean ± SD. Two-way ANOVA with Tukey’s multiple comparisons tests. DC-T cell culture was performed in duplicate. *P < 0.05, **P < 0.01.


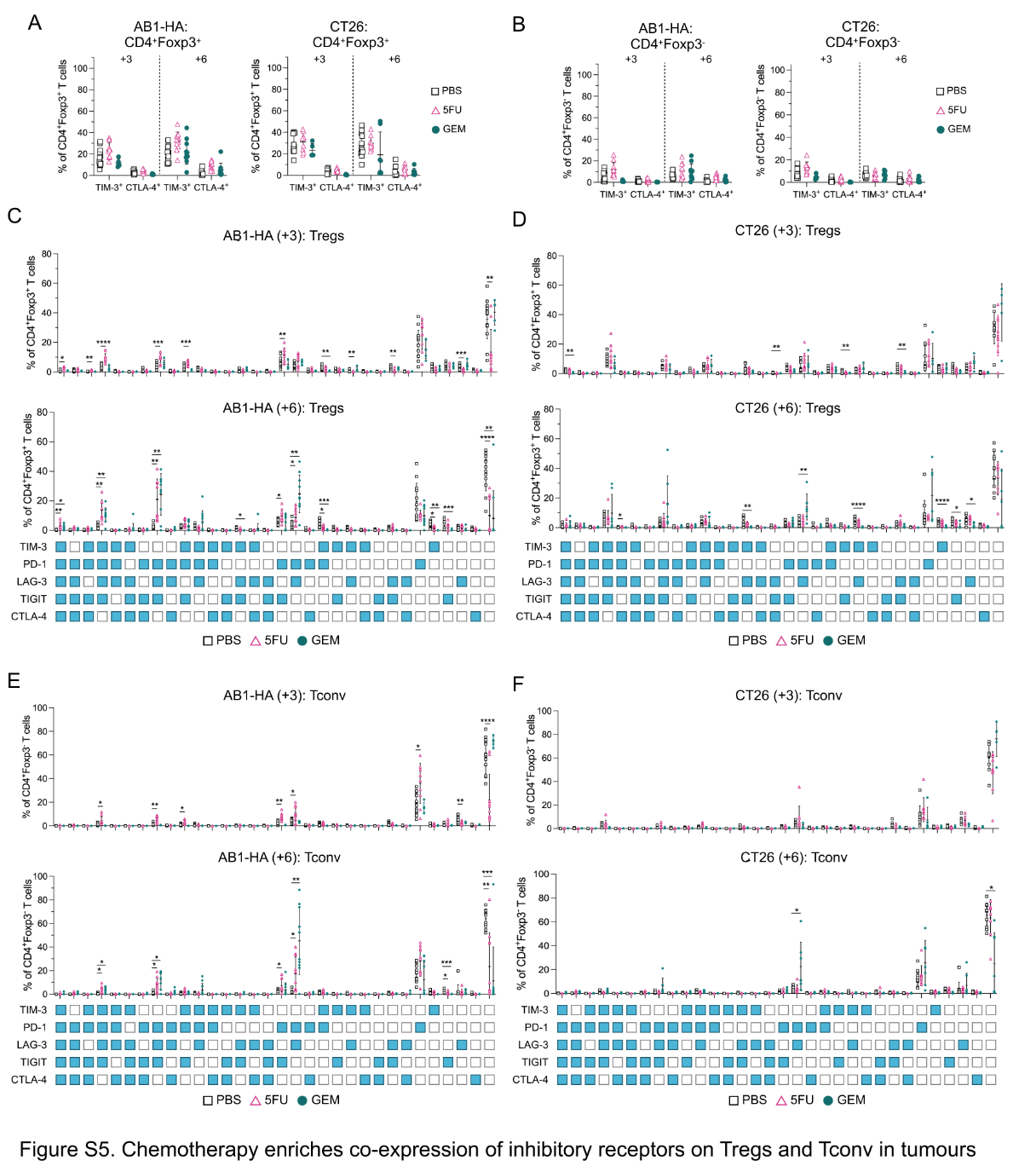


***Figure S5. Co-expression of inhibitory receptors on Tregs and Tconv after anti-metabolite chemotherapy.* (A-B)** Dot plots displaying frequencies of TIM-3 and CTLA-4 on Tregs (CD4^+^Foxp3^+^: **A**) and Tconv (CD4^+^Foxp3^-^: **B**) in AB1-HA (left) and CT26 (right) tumours. **(C-D)** Dot plots showing frequencies of TIM-3, PD-1, LAG-3, TIGIT and CTLA-4 co-expressed on Tregs in AB1-HA **(C)** and CT26 **(D)** tumours after 1 (+3) or 2 (+6) doses of chemotherapy. **(E-F)** Dot plots showing frequencies of TIM-3, PD-1, LAG-3, TIGIT and CTLA-4 co-expressed on Tconv in AB1-HA **(E)** and CT26 **(F)** tumours after 1 (+3) or 2 (+6) doses of chemotherapy. Positive expression of receptor is denoted blue, negative expression is denoted white. Data represented as mean ± SD. Ordinary two-way ANOVA with Tukey’s multiple comparisons test was used to compare between treatment groups and timepoints. Sample sizes n = 5-10 per treatment group. *P < 0.05, **P < 0.01, ****P ≤ 0.0001.


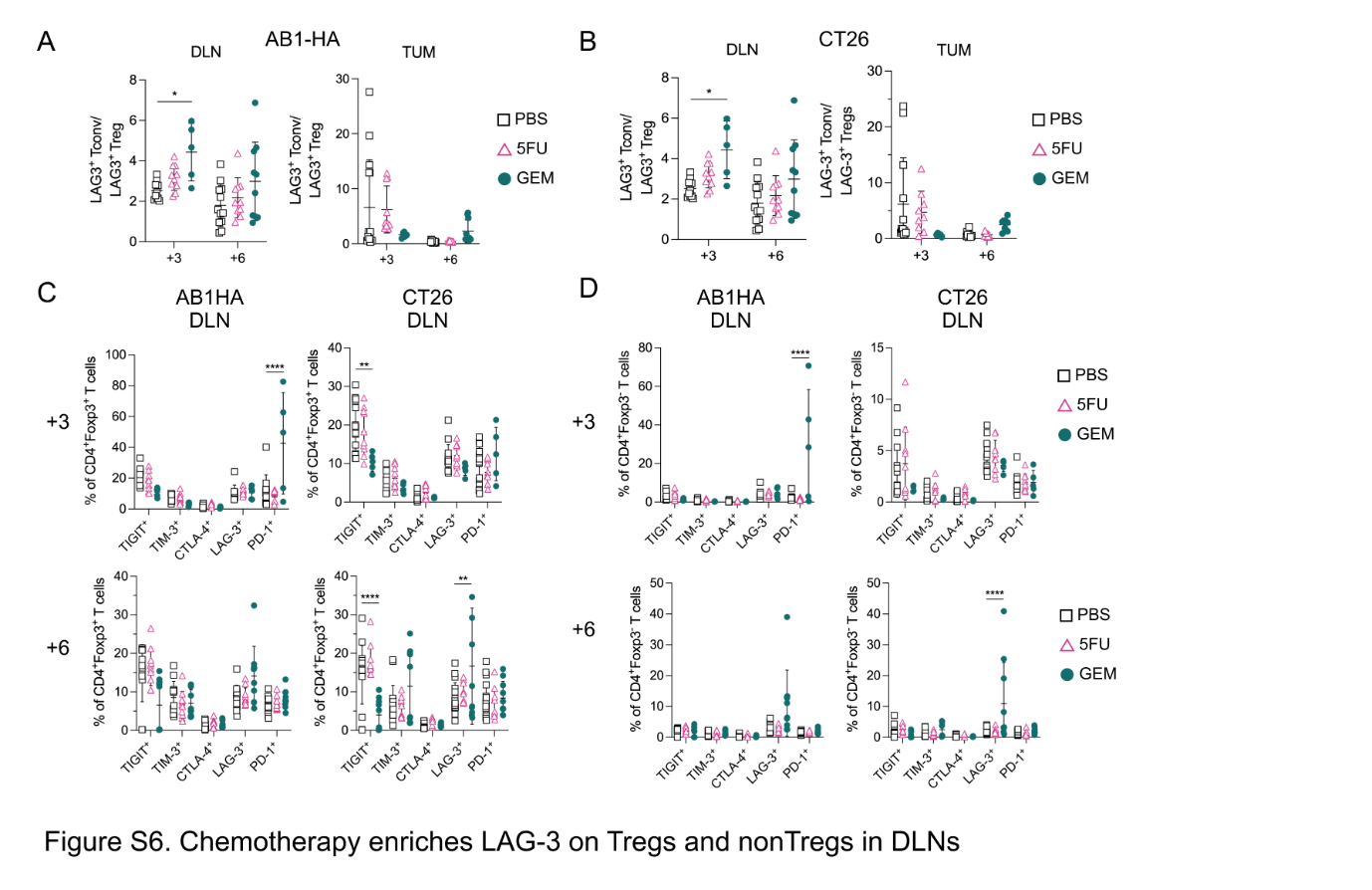


***Figure S6. LAG-3 on Tregs and Tconv in DLNs and tumours* *after anti metabolite chemotherapy* (A-B)** Ratio of LAG-3^+^ Tconv over LAG-3^+^ Tregs in tumours **(A)** and DLNs **(B)** after chemotherapy. **(C-D)** Dot plots displaying the proportion of Tregs (CD4^+^Foxp3^+^; **C**) and Tconv (CD4^+^Foxp3^-^; **D**) expressing inhibitory receptors after 1 (+3) or 2 (+6) doses of chemotherapy in DLNs from AB1-HA and CT26 models. Data represented as mean ± SD. Ordinary two-way ANOVA with Tukey’s multiple comparisons test was used to compare between treatment groups and timepoints. Sample sizes n = 5-10 per treatment group. *P < 0.05, **P < 0.01, ****P ≤ 0.0001.


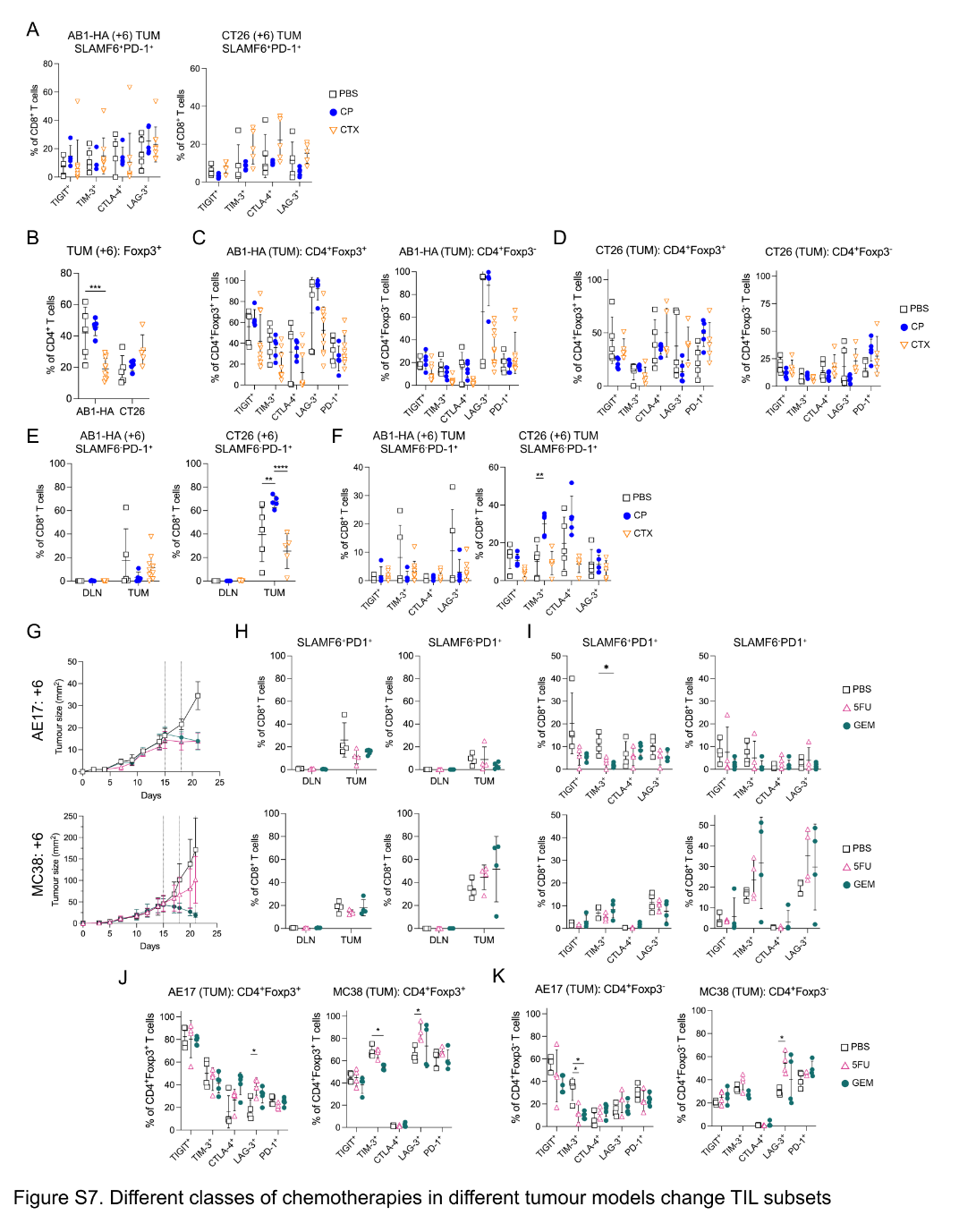


***Figure S7. Effect of different chemotherapies in various tumour models on TIL subsets.* (A)** Dot plots showing the proportions of inhibitory receptors on SLAMF6^+^PD-1^+^ CD8^+^ T_PEX_ after 2 (+6) doses of cisplatin (CP) or cyclophosphamide (CTX) chemotherapy in AB1-HA (left) and CT26 (right) tumours. **(B-D)** Dot plots displaying frequencies of Foxp3^+^ **(B)**, proportion of Tregs and Tconv expressing inhibitory receptors after 2 doses of CP or CTX in AB1-HA **(C)** or CT26 **(D)** tumours. **(E-F)** Dot plots showing percentage of total **(E)** and inhibitory receptor expression **(F)** on SLAMF6^-^PD-1^+^ CD8^+^ T cells after two doses of CP or CTX in AB1-HA (left) and CT26 (right). **(G)** Mean tumour growth curves of AE17 (top) and MC38 (bottom) with two doses of 5FU or GEM chemotherapy. Tumours and DLNs were harvested 3 days after last dose of chemotherapy (+6) for flow cytometry. Dotted lines indicate administration of chemotherapy. **(H-I)** Dot plots displaying frequencies of total **(H)** and inhibitory receptor expression (**I)** on SLAMF6^+~~-~~^PD-1^+^ (left) SLAMF6^-^PD-1^+^ (right) CD8^+^ T cells in AE17 (top) and MC38 (bottom) tumours. **(J-K)** Dot plots displaying proportion of Tregs **(J)** and Tconv **(K)** expressing inhibitory receptors after 2 doses of 5FU or GEM chemotherapy in AE17 (left) or MC38 (right) tumours. Data represented as mean ± SD. Two-way ANOVA with Tukey’s multiple comparisons test was used to compare between treatment groups and timepoints/cell types. Sample sizes for flow cytometry experiments was n = 4-10 per treatment group. *P < 0.05, **P < 0.01, ****P ≤ 0.0001.


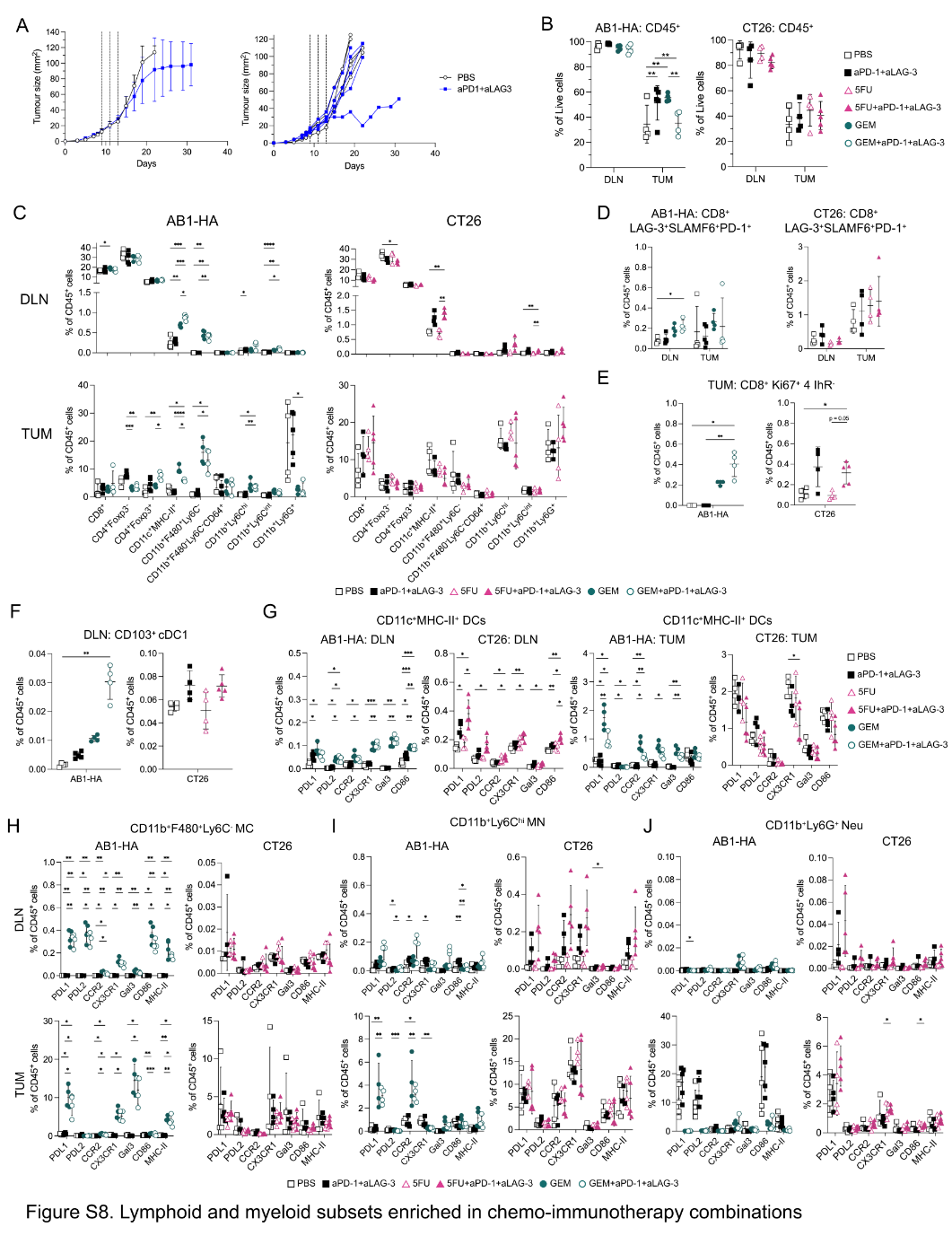


***Figure S8. Lymphoid and myeloid subsets in chemo-immunotherapy combinations* (A)** Tumour growth curves of AB1-HA tumour bearing animals administered with aPD-1+aLAG-3 ICB or PBS when tumours were 9-20mm^2^ in size. (n=5 per group, one experiment). **(B)** Dot plots showing frequency of CD45+ cells in AB1-HA (left) and CT26 (right) chemo-immunotherapy models. **(C)** Dot plots presenting proportions of lymphoid and myeloid subsets in DLNs (top) and tumours (bottom) from AB1-HA (left) and CT26 (right) chemo-immunotherapy models. **(D)** Dot plots displaying frequency of LAG-3^+^SLAMF6^+^PD-1^+^ CD8^+^ T cells in AB1-HA (left) and CT26 (right) chemo-immunotherapy models. **(E)** Dot plots showing frequency of CD8^+^ Ki67^+^ 4IhR^-^ (TIGIT^-^TIM-3^-^LAG-3^-^PD-1^-^CTLA-4^-^) in AB1-HA (left) and CT26 (right) chemo-immunotherapy treated tumours. **(F)** Dot plots showing percentage of CD103^+^ cDC1 (CD11b^-^XCR1^+^) in DLNs from AB1-HA (left) and CT26 (right). **(G)** Dot plots displaying proportions of activation and inhibitory receptors on CD11c^+^MHC-II^+^ dendritic cells from AB1-HA and CT26 DLNs (left) and tumours (right). **(H-J)** Dot plots displaying proportions of activation and inhibitory receptors on CD11b^+^F480^+^Ly6C^-^ macrophages **(H)**, CD11b^+^Ly6C^hi^ monocytes **(I)** and CD11b^+^Ly6G^+^ neutrophils **(J)** from DLN (top) and tumours (bottom) from AB1-HA (left) and CT26 (right) models. *P < 0.05, **P < 0.01, ***P < 0.001, ****P ≤ 0.0001.
